# Spermidine Is an Intercellular Signal Modulating T3SS Expression in Pseudomonas aeruginosa

**DOI:** 10.1128/spectrum.00644-22

**Published:** 2022-04-18

**Authors:** Qiqi Lin, Huishan Wang, Jiahui Huang, Zhiqing Liu, Qunyi Chen, Guohui Yu, Zeling Xu, Ping Cheng, Zhibin Liang, Lian-Hui Zhang

**Affiliations:** a Guangdong Province Key Laboratory of Microbial Signals and Disease Control, Integrative Microbiology Research Center, South China Agricultural University, Guangzhou, China; b Guangdong Laboratory for Lingnan Modern Agriculture, Guangzhou, China; c Institute of Plant Health, Zhongkai University of Agriculture and Engineering, Guangzhou, China; Emory University School of Medicine

**Keywords:** spermidine, *exsCEBA*, T3SS, regulation, quorum sensing, spermidine

## Abstract

Pseudomonas aeruginosa is a vital opportunistic human bacterial pathogen that causes acute and chronic infections. In this study, we set to determine whether the endogenous spermidine biosynthesis plays a role in regulation of type III secretion system (T3SS). The results showed that deletion of *speA* and *speC*, which encode putrescine biosynthesis, did not seem to affect cellular spermidine level and the T3SS gene expression. In contrast, mutation of *speD* and *speE* encoding spermidine biosynthesis led to significantly decreased spermidine production and expression of T3SS genes. We also showed that endogenous spermidine could auto-induce the transcriptional expression of *speE* and its full functionality required the transporter SpuDEFGH. Cytotoxicity analysis showed that mutants Δ*speE* and Δ*spuE* were substantially attenuated in virulence compared with their wild-type strain PAO1. Our data imply a possibility that spermidine biosynthesis in P. aeruginosa may not use putrescine as a substrate, and that spermidine signaling pathway may interact with other two T3SS regulatory mechanisms in certain degree, i.e., cAMP-Vfr and GacS/GacA signaling systems. Taken together, these results specify the role of endogenous spermidine in regulation of T3SS in P. aeruginosa and provide useful clues for design and development antimicrobial therapies.

**IMPORTANCE** Type III secretion system (T3SS) is one of the pivotal virulence factors of Pseudomonas aeruginosa responsible for evading phagocytosis, and secreting and translocating effectors into host cells. Previous studies underline the complicated and elaborate regulatory mechanisms of T3SS for the accurate, fast, and malicious pathogenicity of P. aeruginosa. Among these regulatory mechanisms, our previous study indicated that the spermidine from the host was vital to the host-pathogen interaction. However, the role of endogenous spermidine synthesized by P. aeruginosa on the regulation of T3SS expression is largely unknown. Here we reveal the role and regulatory network of endogenous spermidine synthesis in regulation of T3SS and bacterial virulence, showing that the spermidine is an important interspecies signal for modulating the virulence of P. aeruginosa through regulating T3SS expression.

## INTRODUCTION

Pseudomonas aeruginosa is a notorious opportunistic bacterial pathogen which can cause various acute and chronic infections in immunocompromised individuals (1–3). To survive and maintain its lifestyle, P. aeruginosa can fine-tune gene expression at different stages by sensing and responding to environmental changes (4, 5). As such, the pathogen can assemble multifactorial virulence mechanisms under the control of complicated and sophisticated regulatory systems during the course of pathogen-host interactions. It has been documented that with these regulatory systems, P. aeruginosa could accurately and timely modulate the production of various virulence factors, including flagella, type IV pili, type III secretion system (T3SS), exotoxin A, exoenzymes, elastase, rhamnolipids, phenazines, pyocyanin, lipopolysaccharides, P. aeruginosa lectins (PA), and siderophores, at different stages of infections, which are essential for the establishment and conversion between acute infection and chronic infection (6–8).

Acute infections are symbolized by motility and expression of the T3SS genes, thus T3SS appears to be a highly attractive target for innovative therapies against acute infections (9, 10). T3SS is an assembled injectisome that spans the cell envelope to form a needle-like channel to translocate effector proteins into host cells (11–13). P. aeruginosa contains about 43 T3SS genes, which encode secretion and translocation machinery, effectors (ExoS, ExoT, ExoU, ExoY) and effector-specific chaperones (14, 15). T3SS gene expression is concurrently regulated by a range of intrinsic and extrinsic regulators (16). Among them, ExsA is the master regulator of T3SS in P. aeruginosa, whose activation and expression are closely coupled to a cascade of three interacting proteins, i.e., ExsC, ExsD, and ExsE (17). As an anti-activator, ExsD binds to ExsA and curbs the transcription of ExsA-dependent T3SS genes, and ExsC acts as an anti-anti-activator by binding and restraining ExsD. Under inducing conditions, ExsE is secreted to release ExsC, which then sequesters ExsD and liberates ExsA. Free ExsA binds to the promoters of T3SS genes and thus activates T3SS transcription (18–21). On top of the central regulatory cascade ExsCEBA, several regulatory mechanisms governing T3SS expression have been unveiled in the last few decades. The first one is represented by the global regulator Vfr, which couples with cyclic AMP (cAMP) to form cAMP-Vfr system (CVS) and regulates *exsA* transcription by binding to the promoter of *exsA* (22–24). RNA-binding protein RsmA positively regulates T3SS through the control of *vfr* expression and translation of ExsA (25–28). The second T3SS regulatory mechanism involves multiple components including the two-component system GacS/A, small RNA RsmY/Z, and RNA-binding protein RsmA. RetS positively regulates T3SS via the GacA/S two-component system, which activates the transcription of small RNA RsmY and RsmZ (29–31). RsmY/Z influence T3SS expression by sequestering RsmA when LadS is activated by extracellular calcium (32). The third T3SS regulatory mechanism is associated with the host signal spermidine. The T3SS gene expression of P. aeruginosa was significantly induced by the spermidine signal from mammalian host. Null mutation of the spermidine-specific ABC transporter SpuDEFGH drastically decreased the transcriptional expression of T3SS genes (33). R101-SPM, a spermidine derivative counteracting the functionality of SpuDEFGH, showed a potent activity in inhibition of T3SS expression and the virulence of P. aeruginosa (34).

Spermidine is a polyamine molecule ubiquitously present in prokaryotic and eukaryotic cells at millimolar concentrations (35). Similar to other bacterial organisms, P. aeruginosa is also able to synthesize spermidine and other polyamine molecules (35, 36), but the roles of these endogenous synthesized polyamines on regulation of T3SS gene expression have not yet been characterized. In this study, we aim to determine whether the P. aeruginosa endogenous polyamine biosynthesis and transportation mechanisms could affect T3SS gene expression, and if yes, whether spermidine signaling pathway could interact or cross talk with other T3SS regulatory mechanisms. The related polyamine biosynthesis and transporter genes were knocked out for functional evaluation. The results showed that endogenous spermidine biosynthesis is critical for T3SS regulation, and spermidine transporter SpuDEFGH is required for auto-induction of spermidine biosynthesis and for the full functionality of endogenous spermidine signal in modulating T3SS expression in P. aeruginosa.

## RESULTS

### Effect of the genes encoding polyamine biosynthesis on T3SS gene expression.

**FIG 1:**
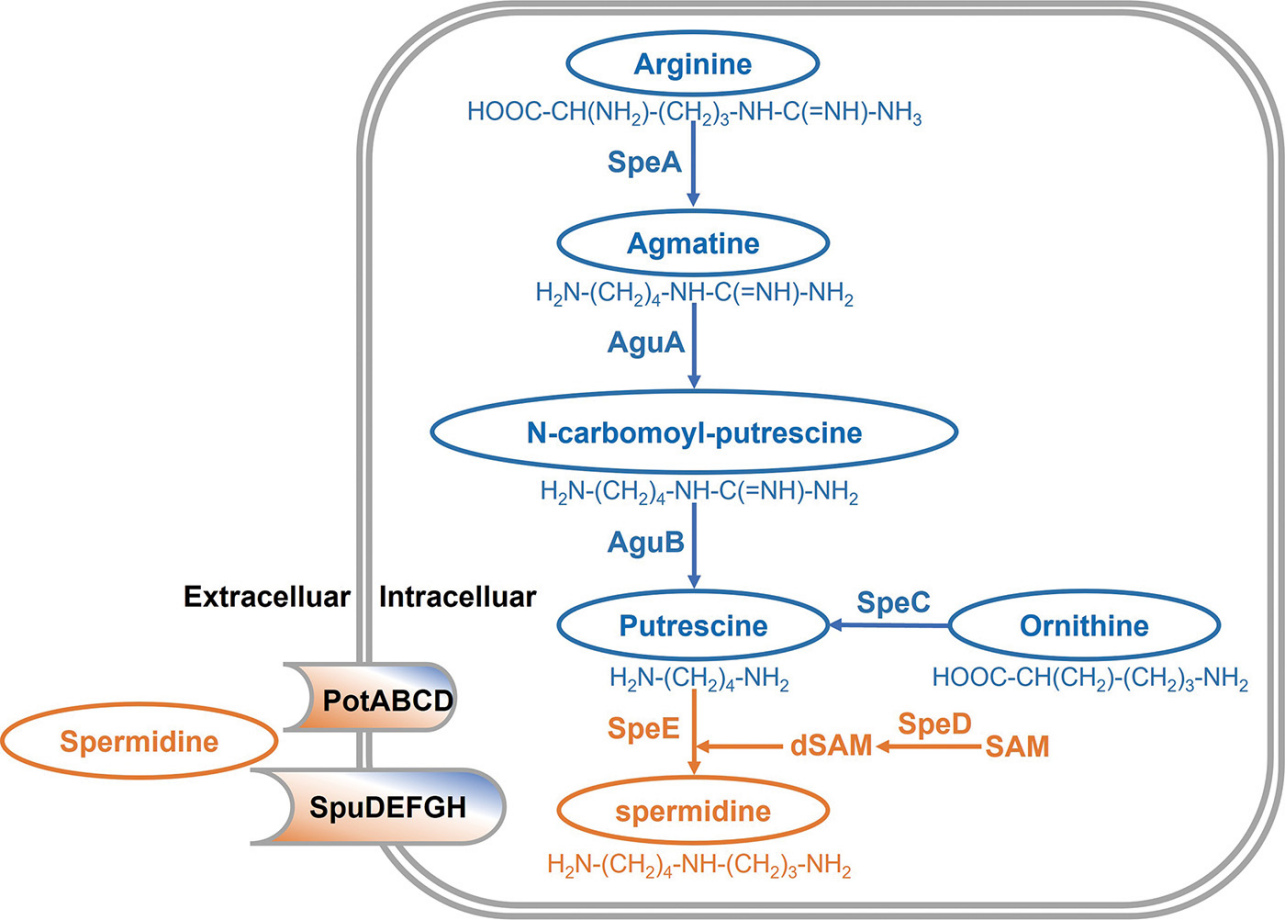
Biosynthesis of putrescine and spermidine in Pseudomonas aeruginosa. AguA, agmatine deiminase; AguB, N-carbamoylputrescine aminotransferase; SpeA, arginine decarboxylase; SpeC, ornithine decarboxylase; SpeD, SAM decarboxylase; SpeE, spermidine synthase; SpuDEFGH, spermidine ABC transporter substrate-binding protein; PotABCD, polyamine ABC transporter substrate-binding protein; SAM, S-adenosyl methionine; dSAM, decarboxylated SAM.

Polyamines are low molecular weight aliphatic nitrogenous bases containing two or more amino groups, including putrescine, cadaverine, spermidine, spermine, and so on. In P. aeruginosa, putrescine can be synthesized either from arginine through the arginine decarboxylase SpeA pathway or from ornithine decarboxylation pathway catalyzed by SpeC (37–39). SpeC catalyzes one-step reaction from ornithine to putrescine, whereas multiple reactions are required in the arginine decarboxylation pathway. Arginine is firstly converted to agmatine by SpeA, and then agmatine deiminase AguA catalyzes formation of N-carbamoyl-putrescine, which is further converted into putrescine by amidinohydrolase AguB. Spermidine is synthesized from putrescine by SpeE by addition of the aminopropyl moiety derived from decarboxylated S-adenosylmethionine (dSAM). Formation of dSAM is catalyzed by SpeD (36, 39). Bioinformatics analysis showed that P. aeruginosa genome contains all the above genes involved in spermidine biosynthesis (Fig. 1).

**FIG 2:**
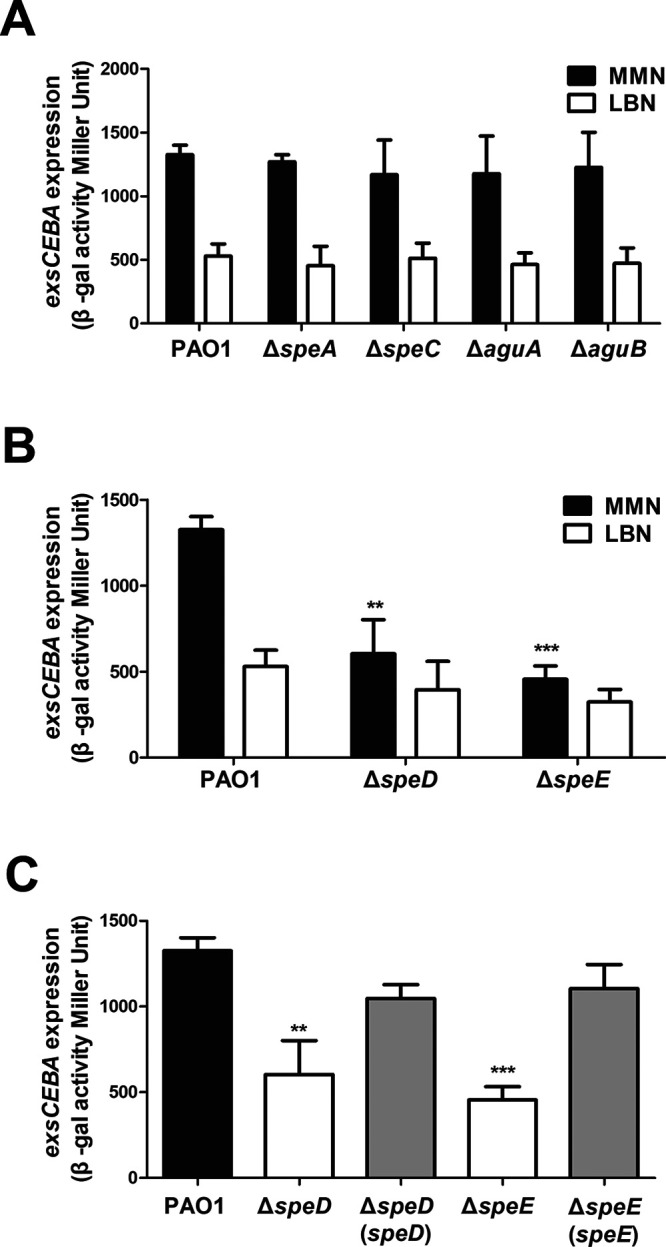
Effect of the genes encoding putrescine and spermidine biosynthesis on expression of T3SS *exsCEBA* genes. (A) Expression patterns of *exsCEBA*-*lacZ* in wild-type strain PAO1 and the mutants of putrescine biosynthesis associated genes. (B) Expression patterns of *exsCEBA*-*lacZ* in wild-type strain PAO1 and the mutants of spermidine biosynthesis associated genes. (C) Expression patterns of *exsCEBA*-*lacZ* in wild-type strain PAO1, spermidine biosynthesis gene mutants, and corresponding complementation strains in MMN medium. Bacterial strains were cultured in different growth media as indicated. The experiment was repeated at least triplicates in each assay and error bars indicate standard deviations. Statistics significance: **, *P < *0.01; ***, *P < *0.001. Statistical analysis compared to wild-type strain PAO1 in the same medium was performed by using Student's *t* test.

To determine whether the above spermidine biosynthesis genes contribute to regulation of T3SS in P. aeruginosa, the promoter of *exsCEBA* operon was fused to the reporter gene *lacZ* and the construct pClacZ was integrated into the chromosome of P. aeruginosa to obtain the T3SS reporter strain PAO1pClacZ (pCZ) as described previously (33). In order to verify the roles of putrescine and spermidine biosynthesis genes in modulation of T3SS, the in-frame deletion mutants of *speA*, *aguA*, *aguB*, *speC*, *speD*, and *speE* genes were generated using the reporter pCZ as the parental strain. We found that deletion of these genes did not affect the growth of P. aeruginosa PAO1 in the different media used in this study (Fig. S1). Quantitative *β*-galactosidase assay analysis showed that mutation of the putrescine biosynthesis genes *speA*, *speC*, *aguA*, and *aguB* did not cause significant changes on the expression level of *exsCEBA* either in LB supplemented with NTA (LBN) or in the minimal medium (MM) supplemented with NTA (MMN) (Fig. 2A), suggesting that putrescine biosynthesis pathway is not essential in regulation of T3SS expression. In contrast, mutation of spermidine biosynthesis genes *speD* and *speE* caused a significant reduction in the expression level of *exsCEBA* in MMN medium (Fig. 2B), which was restored by complementation of corresponding genes in the mutants (Fig. 2C). This result unveils the roles of spermidine biosynthesized genes in modulating the expression of T3SS genes. The contribution of the spermidine synthesized by *speD* and *speE* on the regulation of T3SS was also evident. As expected in LBN medium, which harbors the polyamine molecules with spermidine (33), deletion of *speD* and *speE*, respectively, caused much less reduction in expression level of *exsCEBA* than in the MMN medium without polyamine molecules (Fig. 2B), which suggests the spermidine in LBN medium can rescue the defective production of spermidine in mutants Δ*speD* and Δ*speE.*

### Effect of the genes encoding polyamine transportation on T3SS gene expression.

**FIG 3:**
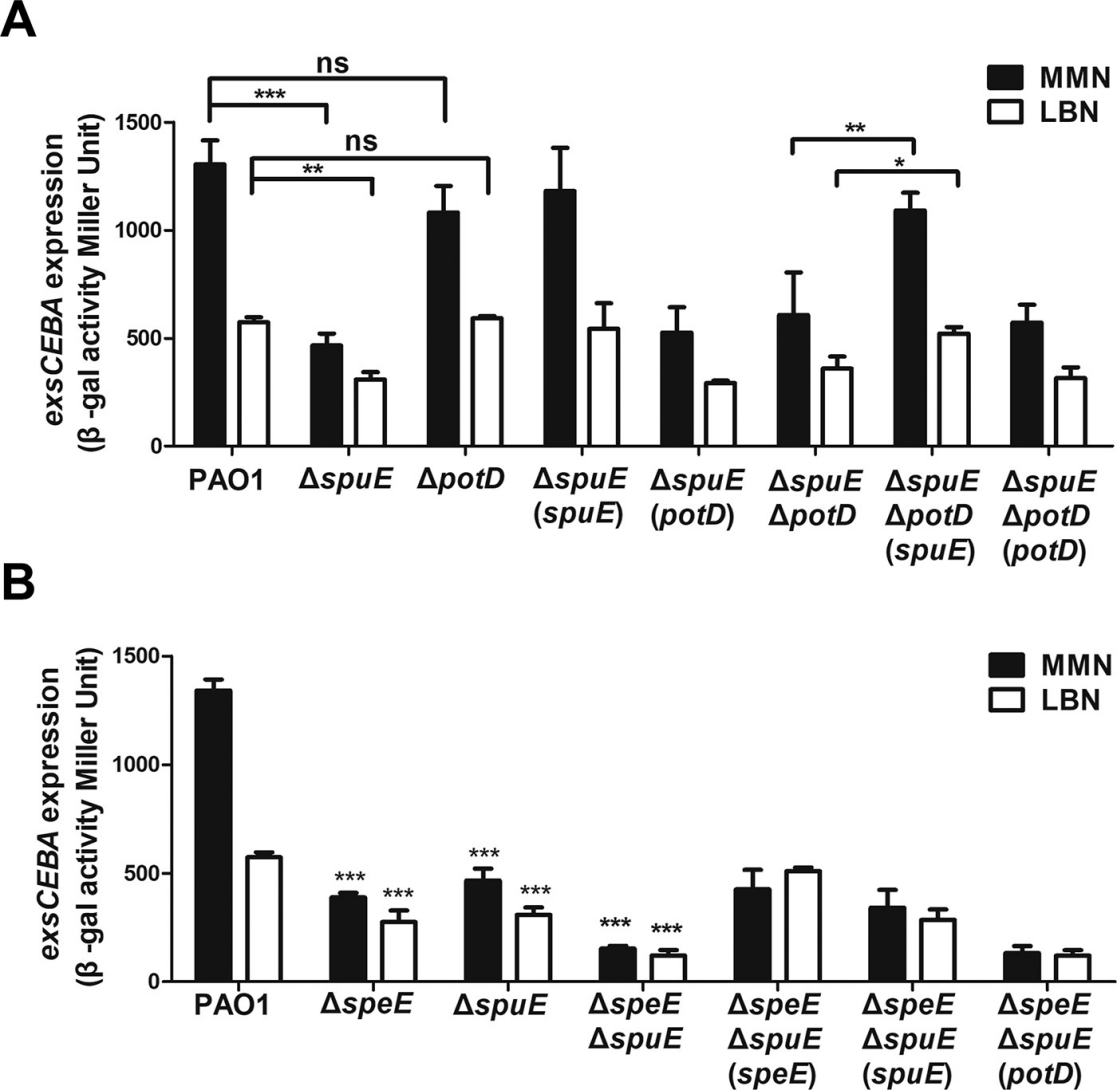
Effect of the genes encoding polyamine transportation on expression of T3SS *exsCEBA* genes. (A) Impact of *spuE*-*potD* double deletion and with corresponding overexpression on *exsCEBA* expression. (B) Impact of *spuE*-*speE* double deletion and with corresponding overexpression on *exsCEBA* expression. Wild-type strain PAO1 and its derivatives were cultured in different growth media as indicated. Experiment was repeated at least triplicates in each assay and error bars indicate standard deviations. Statistics significance: ns, no significance; *, *P < *0.05; **, *P < *0.01; ***, *P < *0.001. Statistical analysis compared to wild-type strain PAO1 or *spuE*-*potD* double deletion mutant in the same medium was performed by using Student’s *t* test.

We were curious whether the membrane transporters are required for the bacterial endogenous spermidine signaling. Bioinformatics analysis showed that P. aeruginosa encodes at least two sets of polyamine transporters, i.e., SpuDEFGH and PotABCD. SpuDEFGH, which is a major spermidine uptake system (40), was shown to play an essential role in response to host or extracellular spermidine signal for modulating T3SS gene expression (33). In contrast, the biological functions of PotABCD in P. aeruginosa is largely unknown. Sequence alignment showed that SpuE and PotD, which are the substrate binding proteins of the corresponding transporters, share about 35.2% similarity at amino acid level with each other. Besides, the SpuE and PotD in P. aeruginosa also share about 37.9% and 32.1% similarity, respectively, compared with the spermidine-binding protein PotD in Escherichia coli (41) (Fig. S2). To investigate whether transporter PotABCD also plays a role in regulation of T3SS in response to polyamine signals, we performed single deletion in *spuE* and *potD* genes, respectively, in strain pCZ for analyzing their impact on the activity of *exsCEBA* promoter by *β*-galactosidase analysis. We found that deletion of these genes did not affect the bacterial growth in comparison with wild-type strain PAO1 (Fig. S1A, C, and E). The result of *β*-galactosidase analysis indicated that expression of *exsCEBA* in mutant Δ*spuE* was decreased in both MMN and LBN media compared with the wild-type strain PAO1 as reported previously (33), whereas expression of *exsCEBA* in mutant Δ*potD* did not show significant change in both MMN and LBN media compared with those of wild-type strain PAO1 (Fig. 3A). *In trans* expression of the *spuE* in Δ*spuE* restored the *exsCEBA* expression. However, expressing the *potD* *in trans* in Δ*spuE* had no effect on *exsCEBA* expression (Fig. 3A). To test the synergistic effect of PotABCD and SpuDEGHF on modulating the T3SS expression, the double-deletion mutant Δ*spuE*Δ*potD*, single-complemented strains Δ*spuE*Δ*potD* (*spuE*), and Δ*spuE*Δ*potD* (*potD*) were constructed. The results indicated that the level of *exsCEBA* expression in double mutant Δ*spuE*Δ*potD* and the single deletion mutant Δ*spuE* were comparable in both MMN and LBN media (Fig. 3A). As expected, complementation of Δ*spuE*Δ*potD* with *spuE* restored *exsCEBA* expression to a level as that in Δ*potD*, but *in trans* expression of *potD* in Δ*spuE*Δ*potD* did not have similar effect on *exsCEBA* expression neither in MMN nor in LBN (Fig. 3A). The above results suggest that PotABCD transporter is not associated with the spermidine signaling regulation of T3SS in P. aeruginosa.

### Null mutation of both spermidine biosynthesis and transportation genes abrogates T3SS expression.

To evaluate the impact of deleting both spermidine synthase and transporter genes on T3SS expression in P. aeruginosa, we constructed the double deletion mutants Δ*speD*Δ*potD* and Δ*speE*Δ*spuE* by deleting *potD* and *spuE* using the single mutants Δ*speD* and Δ*speE* as parental strains, respectively. Quantitative *β*-galactosidase assay in MMN medium showed that overexpression of either *spuE* or *potD* in the double mutant Δ*speD*Δ*potD* did not cause any significant changes on *exsCEBA* expression (Fig. S3). In contrast, as shown in Fig. 3B, double deletion mutant Δ*speE*Δ*spuE* has a further reduction on expression level of *exsCEBA* in either LBN or MMN media compared with the single mutants Δ*speE* and Δ*spuE*. Complementation of the double mutant Δ*speE*Δ*spuE* with the spermidine synthase gene *speE* restored *exsCEBA* expression to a level of that in the single mutant Δ*spuE* in MMN and increased *exsCEBA* expression to a level of that in wild-type strain PAO1 in LBN (Fig. 3B). Besides, *in trans* expression of the spermidine transporter gene *spuE* in the double mutant Δ*speE*Δ*spuE* restored the *exsCEBA* expression to a level of that in Δ*speE* in both media. Consistent with the dispensable role of PotABCD in regulation of *exsCEBA* expression, *in trans* expression of *potD* did not alter the *exsCEBA* expression in mutant Δ*speE*Δ*spuE* (Fig. 3B). These results suggest that both endogenous spermidine biosynthesis and spermidine uptake system are required in regulation of T3SS expression in P. aeruginosa.

### Effect of spermidine and putrescine on *exsCEBA* gene expression.

**FIG 4:**
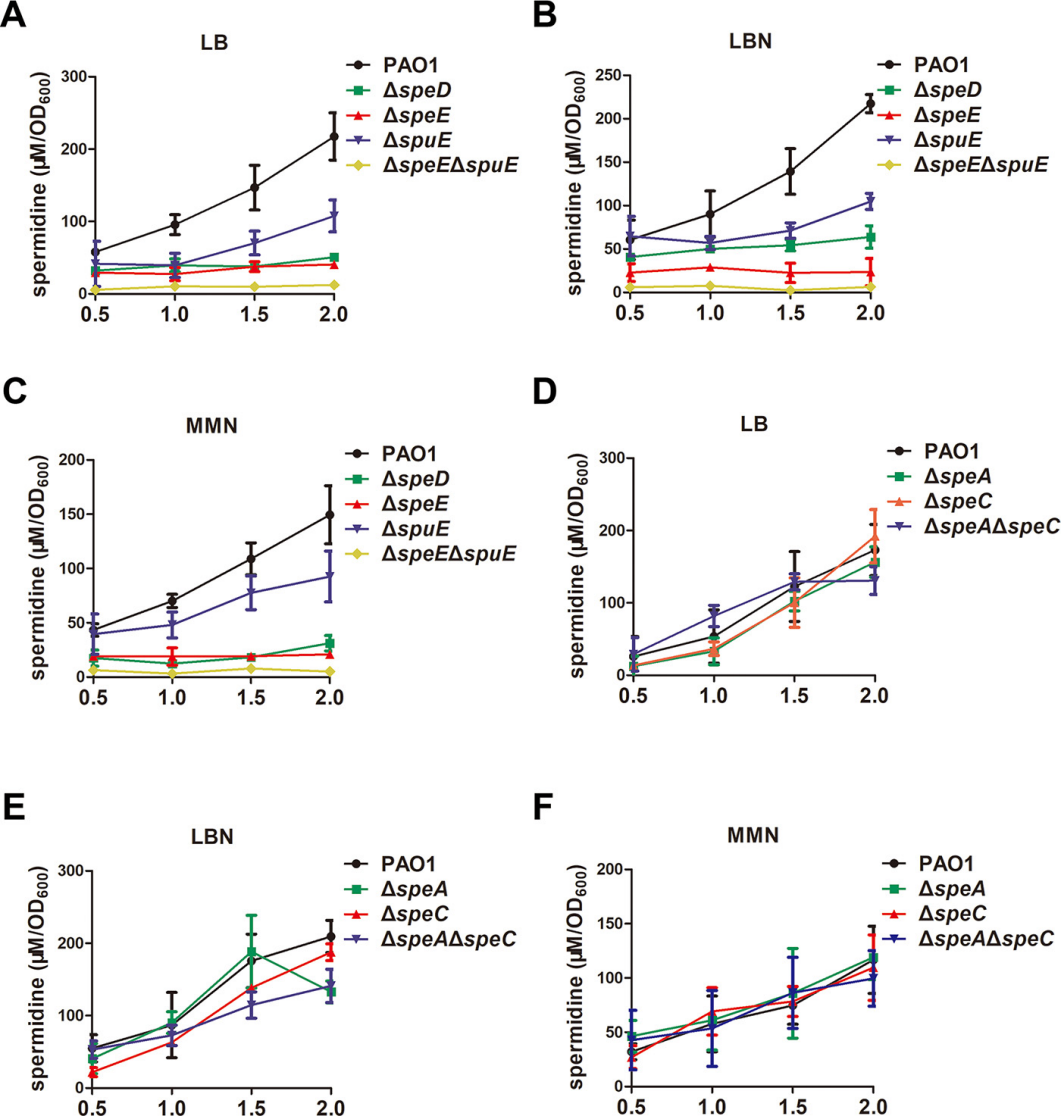
Cellular spermidine levels in Pseudomonas aeruginosa PAO1 and its derivatives. The intracellular spermidine molecules were benzoyled and examined by liquid chromatography coupled with mass spectrometry (LC-MS). The P. aeruginosa PAO1 and derivatives were cultured in LB (A and D), LBN (B and E), and MMN (C and F) as indicated. Bacterial cells were harvested for assay of spermidine levels when bacterial cell density reached OD_600_ of 0.5, 1.0, 1.5, and 2.0, respectively. The experiment was repeated at least triplicates in each assay and error bars indicate standard deviations.

**FIG 5:**
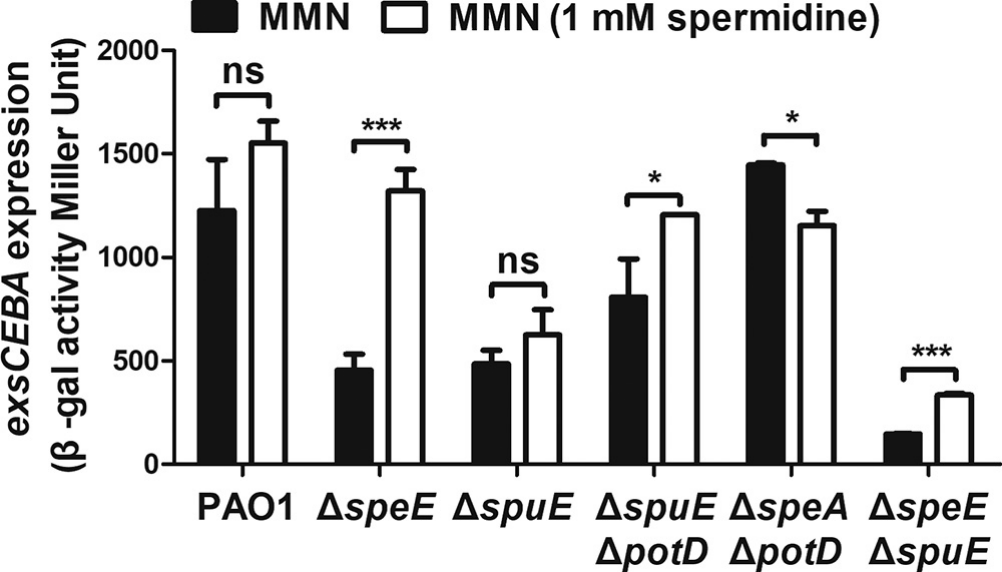
Effect of spermidine on T3SS gene expression. Spermidine was added in MMN at a final concentration of 1 mM to determine the expression level of *exsCEBA* in different bacterial strains. Experiment was repeated at least triplicates in each assay and error bars indicate standard deviations. Statistics significance: ns, no significance; *, *P < *0.05; ***, *P < *0.001. Statistical analysis comparison to wild-type strain PAO1 was performed by using Student’s *t* test.

Deletion of either *speE* or *spuE*, which encodes spermidine biosynthesis and the substrate-binding protein of spermidine transporter, respectively, caused significant reductions in T3SS gene expression (Fig. 2 and 3). We thus compared the intracellular spermidine level in wild-type strain PAO1 and the mutants Δ*speD*, Δ*speE*, and Δ*spuE* in LB, LBN, and MMN medium. LC-MS analysis showed that at the late growth stage (OD_600_ = 2.0), deletion of *spuE*, *speD*, or *speE* reduced the intracellular spermidine level by one third to two third while double deletion of *speE* and *spuE* almost completely abolished the accumulation of spermidine (Fig. 4A to C). Considering that putrescine may serve as the precursor of spermidine (Fig. 1A), we set to determine whether exogenous addition of spermidine and putrescine could rescue the diminished expression of *exsCEBA* in the relevant mutants. Surprisingly, we found that the T3SS gene expression in wild-type strain PAO1 was inhibited by exogenous putrescine in a dosage-dependent manner (Fig. S4A), and similarly, the expression of *exsCEBA* was significantly downregulated with exogenous addition of 1 mM putrescine in P. aeruginosa PAO1 and its derivatives (Fig. S4B). In contrast, addition of spermidine at a final concentration of 1 mM in MMN medium restored the defective expression of *exsCEBA* in the spermidine synthase mutant Δ*speE* (Fig. 5).

### Spermidine biosynthesis is essential for the full virulence of P. aeruginosa.

**FIG 6:**
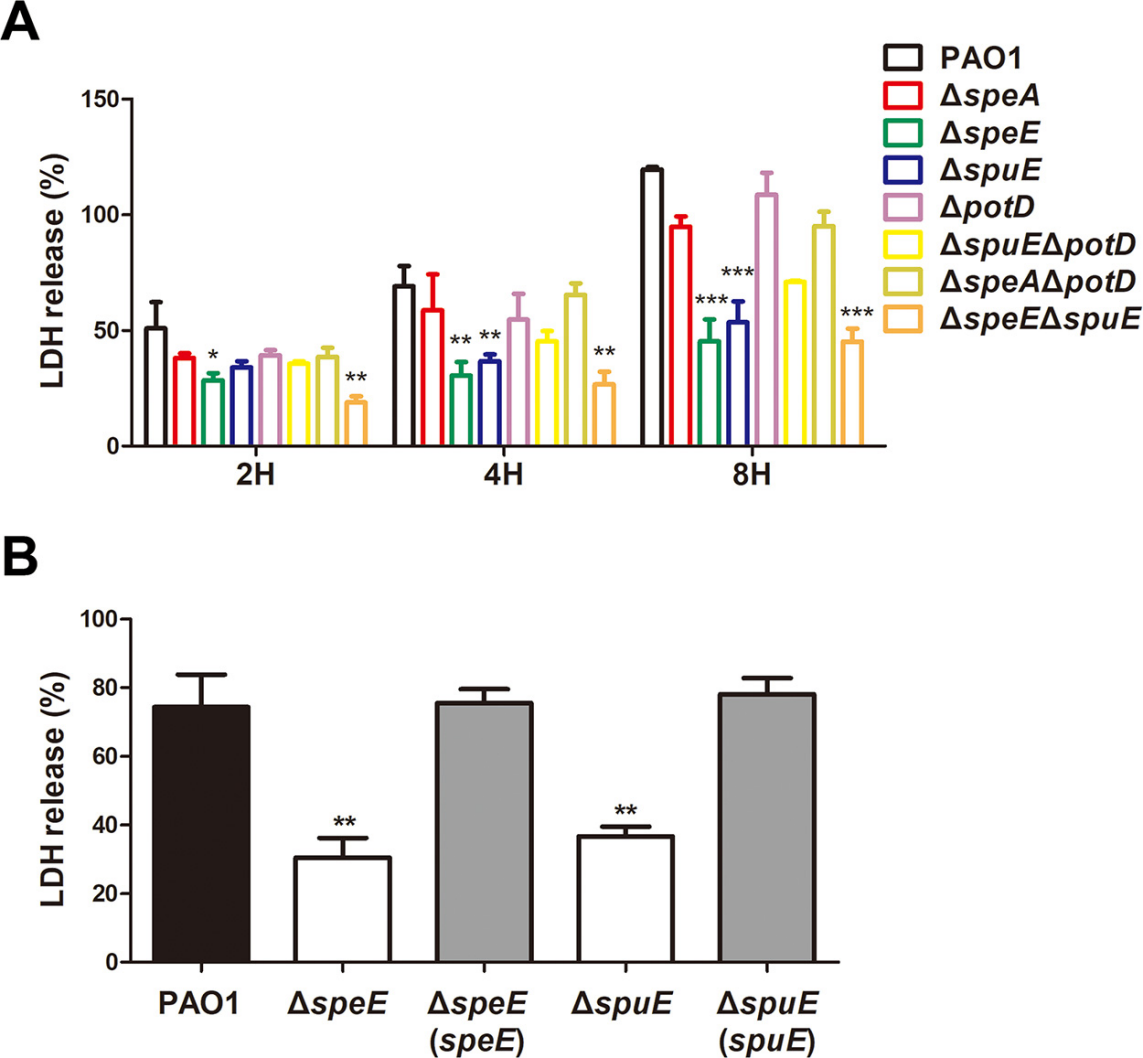
Spermidine biosynthesis is essential for the full virulence of Pseudomonas aeruginosa. (A) Relative LDH released from the cell line A549 challenged with wild-type strain PAO1 and its derivatives at different time points postinoculation. (B) *In trans* expression of *speE* and *spuE* in corresponding mutants restored the cell cytotoxicity. Wild-type strain PAO1 and its derivatives were grown in MMN medium, and the LDH activity was measured 4-h postinoculation. Experiment was repeated at least triplicates in each assay and error bars indicate standard deviations. Statistics significance: *, *P < *0.05; **, *P < *0.01; ***, *P < *0.001. Statistical analysis compared to wild-type strain PAO1 in the same incubation time was performed by using Student’s *t* test.

To determine the role of spermidine biosynthesis on T3SS-dependent virulence in P. aeruginosa, we infected the human lung epithelial cell A549 with wild-type strain PAO1 and its derived mutants Δ*speA*, Δ*speE*, Δ*spuE*, Δ*potD*, Δ*spuE*Δ*potD*, Δ*speA*Δ*potD*, and Δ*speE*Δ*spuE* cultured in MMN medium. As shown in Fig. 6A, compared with bacterial cytotoxicity of the wild-type strain PAO1, bacterial cytotoxicity of mutants Δ*speA*, Δ*potD*, and Δ*speA*Δ*potD* were slightly reduced and those of mutants Δ*speE* and Δ*spuE* were dramatically declined. Consistent with the role of T3SS in establishment of acute infection, compared with wild-type strain PAO1 and other mutants, the spermidine synthesis and transporter mutant Δ*speE*Δ*spuE* had less cytotoxicity at the early infection stages (2 h and 4 h) than at late infection stage (8 h) (Fig. 6A). Complementation of *speE* or *spuE* in corresponding mutants Δ*speE* or Δ*spuE* fully restored the defective cytotoxicity (Fig. 6B).

**FIG 7:**
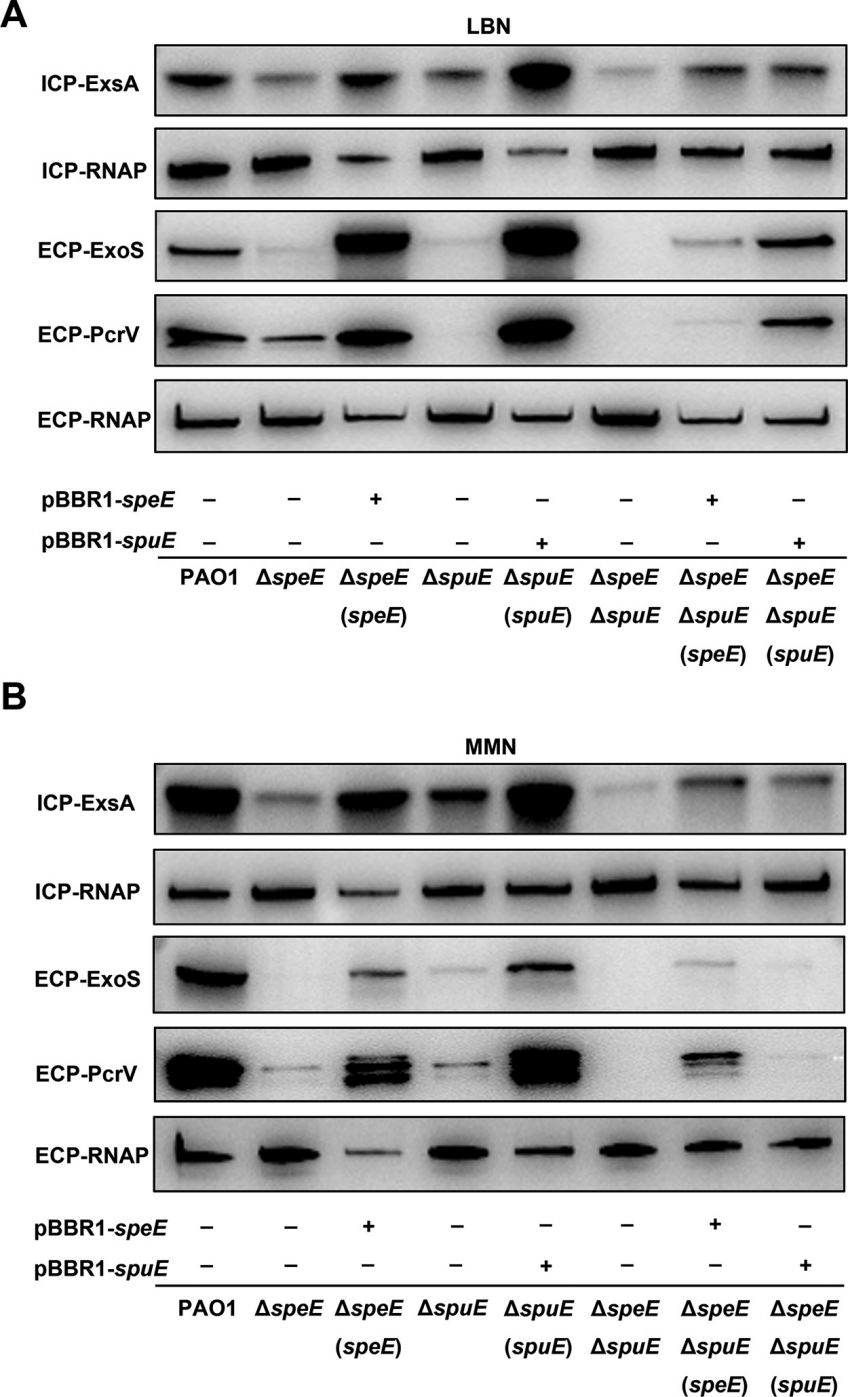
Effect of spermidine biosynthesis and transportation on the expression of T3SS associated protein. Immunoblotting detection of the protein levels of ExsA, ExoS, and PcrV in different strains cultured in LBN medium (A) or MMN medium (B). The cell extracts were subjected to SDS-PAGE separation and for immuno-blotting. RNA polymerase (RNAP) was used as an internal control. Symbol: ECP, extra-cellular proteins; ICP, intra-cellular proteins; + and −, presence (+) or absence (−) of the expression construct in corresponding bacterial strain.

To assess whether the attenuated cytotoxicity was caused by defective T3SS, wild-type strain PAO1 and mutants Δ*speE*, Δ*spuE*, and Δ*speE*Δ*spuE* were grown in MMN and LBN, respectively, and expression of T3SS master regulator ExsA, effector ExoS, and structural secretion protein PcrV were examined by Western blotting analysis. The results showed that the protein level of ExsA, ExoS, and PcrV were substantially reduced or nearly abolished in mutants Δ*speE*, Δ*spuE*, and Δ*speE*Δ*spuE* (Fig. 7). Complementation of the mutants Δ*speE* and Δ*spuE* with corresponding genes fully rescued the defective expression of the three T3SS proteins (Fig. 7), indicating that spermidine biosynthesis enzyme SpeE and transporter SpuE are indispensable to the virulence of P. aeruginosa.

### Spermidine biosynthesis is auto-induced in P. aeruginosa.

**FIG 8:**
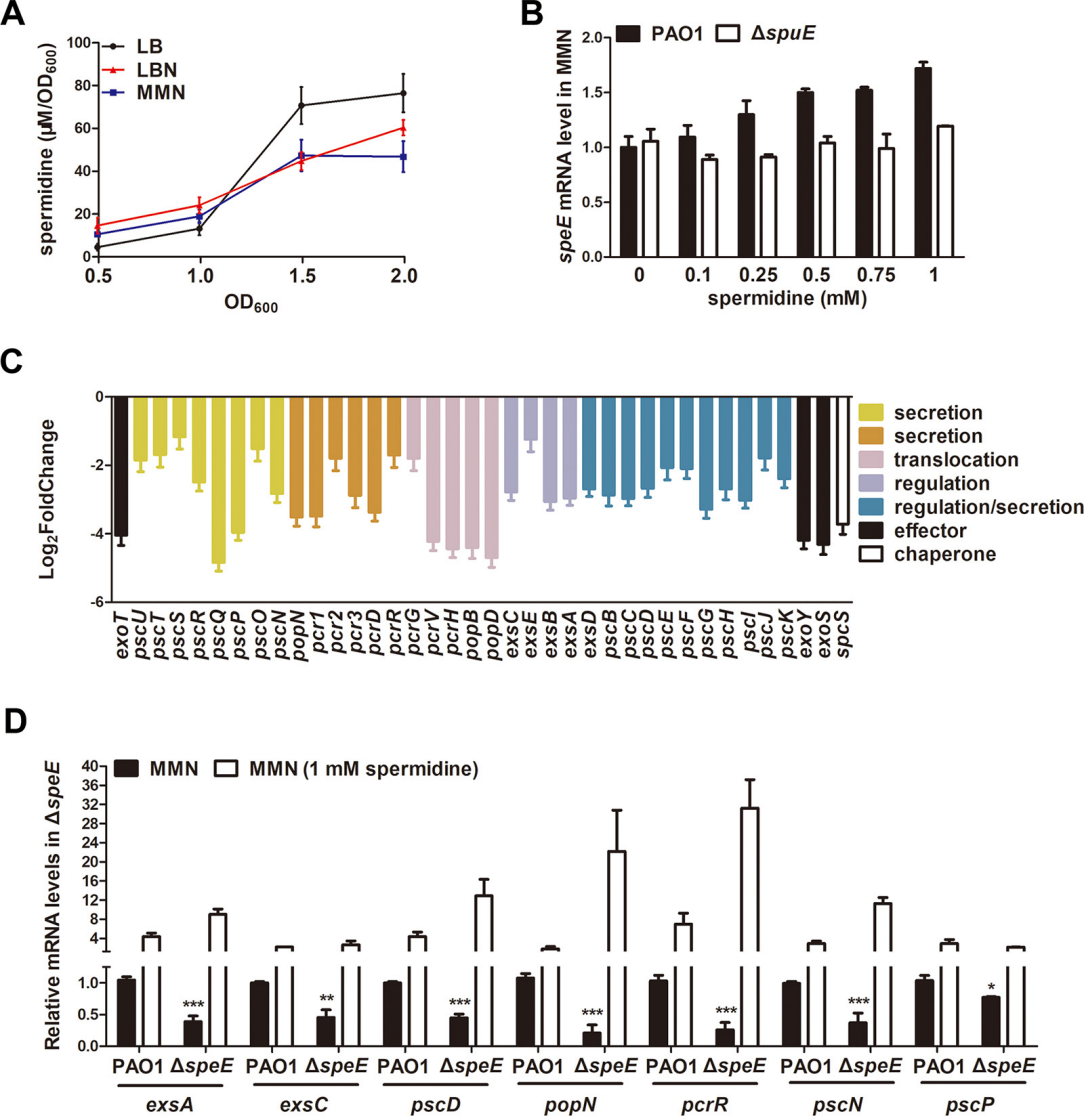
Spermidine biosynthesis pattern and regulatory spectrum. (A) Spermidine levels of wild-type strain PAO1 in different media at different time points. (B) RNA levels of *speE* in wild-type strain PAO1 and mutant Δ*spuE* with or without different concentrations of spermidine. (C) Transcriptome analysis revealed the T3SS genes affected by null mutation of the spermidine synthase gene *speE*. (D) RT-qPCR analysis of the T3SS-related genes affected by null mutation of *speE*. Total RNAs were collected when bacterial strains were grown to an OD_600_ of 1.0 to 1.3 with or without exogenous supplement of spermidine in MMN medium. The *rpoD* gene was used as an internal control. Experiment was repeated at least triplicates in each assay and error bars indicate standard deviations. Statistics significance: *, *P < *0.05; **, *P < *0.01; ***, *P < *0.001. Statistical comparison was performed by using Student's *t* test. Statistical analysis compared of wild-type strain PAO1 and mutant Δ*speE* in MMN was performed by using Student’s *t* test.

To understand the regulatory mechanisms of spermidine in P. aeruginosa, we determined the cellular spermidine levels of wild-type strain PAO1 cultured in LB, LBN, and MMN media, respectively, at different time points via LC-MS. As shown in Fig. 8A, spermidine levels of wild-type strain PAO1 cultured in three media were increasing along with bacterial proliferation. Spermidine levels of wild-type strain PAO1 in LBN and MMN were slightly higher than those in LB at the early stages of bacterial growth (OD_600_ = 0.5 and 1.0) but decreased at the late growth stages (OD_600_ = 1.5 and 2.0) than those in LB medium, suggesting that T3SS-inducing conditions is beneficial to the production of spermidine in P. aeruginosa at early bacterial growth stages. We then tested whether spermidine synthase genes could be auto-induced like other typical quorum sensing genes. The results showed that in MMN medium supplemented with different concentrations of spermidine, the expression level of *speE* in wild-type strain PAO1 was enhanced along with the increasing dosage of spermidine but those in the spermidine transporter mutant Δ*spuE* remains stable (Fig. 8B). Taken together, these findings indicate that the synthesis of spermidine is cell-density dependent, and spermidine can auto-induce its biosynthesis in a way similar to other quorum sensing (QS) signals.

### Regulatory spectrum of spermidine QS system.

To explore the potential regulatory spectrum of SpeE, we examined the global gene expression profiles of wild-type strain PAO1 and the mutant Δ*speE* in MMN medium using transcriptome analysis. Deletion of *speE* significantly decreased the expression of not only the T3SS genes (Fig. 8C; Table S3) but also genes encoding other biological functions, including chemotaxis, flagellar assembly, and metabolism (Fig. S5). A total of 62 and 20 genes were downregulated and upregulated, respectively, in the *speE* mutant compared with the wild-type strain PAO1 (|log_2_ fold change| ≥ 1, *P < *0.05; Table S3). In particular, 38 genes related to T3SS were significantly downregulated in *speE* gene mutant compared with wild-type strain PAO1 (Table S3). We used RT-qPCR to further verify T3SS gene expression in wild-type PAO1 and mutant Δ*speE* with or without exogenous addition of spermidine. As shown in Fig. 8D, the results showed expression of *exsA*, *exsC*, *pcsD*, *popN*, *pcrR*, *pscN*, and *pscP* in mutant Δ*speE* were significantly decreased compared with those in wild-type strain PAO1, which could be recovered by exogenous addition of 1 mM spermidine. These results indicate that SpeE and its product spermidine play important roles in regulating the expression of T3SS.

### Expression patterns of spermidine-related genes.

**FIG 9:**
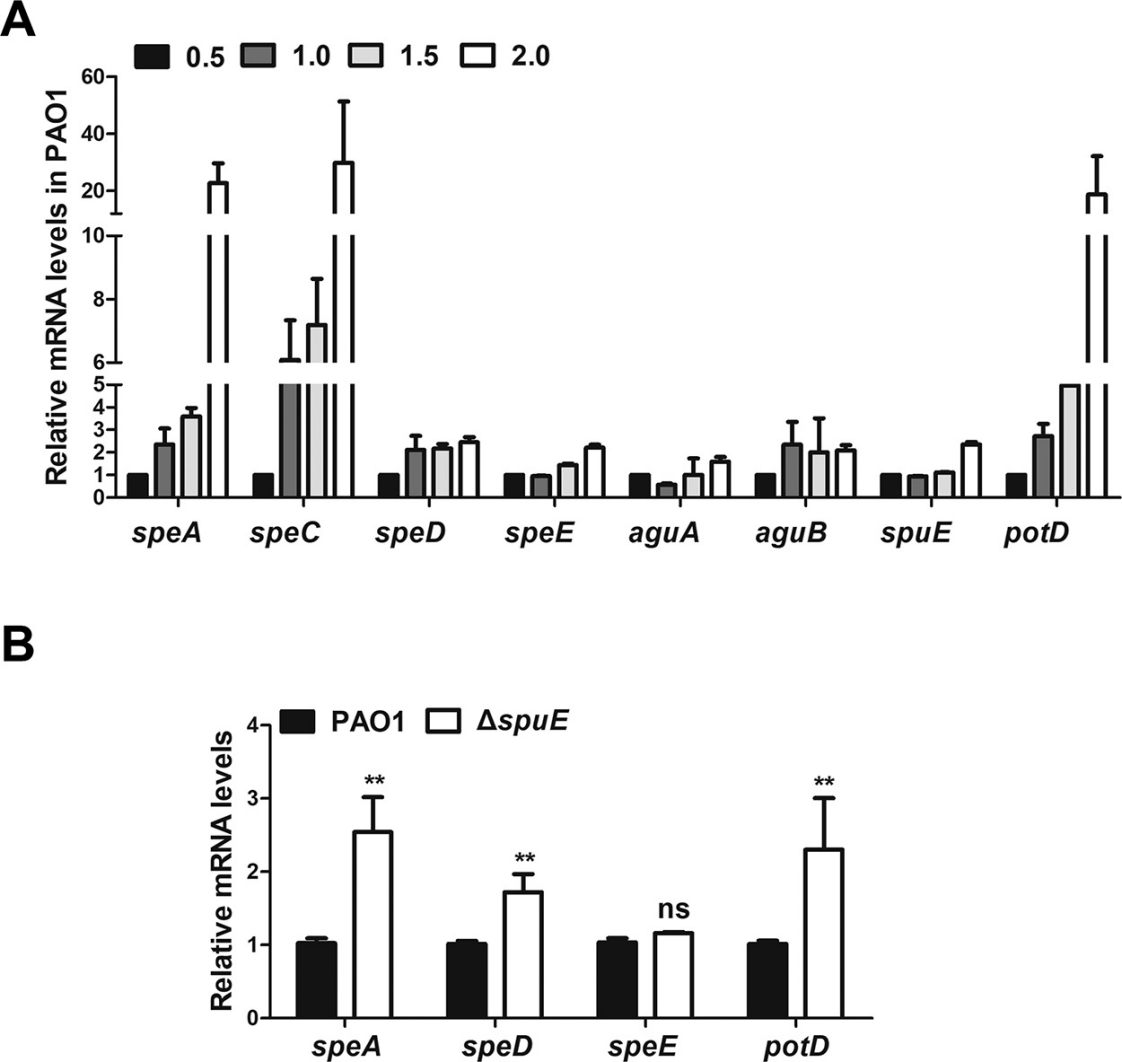
Expression patterns of spermidine-related genes. (A) Time-course analysis of spermidine-related gene expression in wild-type strain PAO1 by RT-qPCR. Total RNAs were collected when bacterial cells were grown to the corresponding OD_600_. (B) Impact of *speE* null mutation on expression of spermidine-related genes. Total RNAs were collected when bacterial cells were grown to an OD_600_ of 1.2. The *rpoD* gene was used as an internal control. Each assay was conducted with at least triplicates and error bars indicate standard deviations. Statistics significance: ns, no significance; **, *P < *0.01. Statistical analysis comparison to wild-type strain PAO1 was performed by using Student’s *t* test.

To determine the expression patterns of the genes associated with spermidine biosynthesis and transportation, we monitored the transcript levels of *speA*, *speC*, *aguA*, *aguB*, *speD*, *speE*, *potD*, and *spuE* in wild-type strain PAO1 in MMN medium at OD_600_ = 0.5, 1.0, 1.5, and 2.0, respectively. The results indicated that the transcriptional expression of all the tested genes were ascended at the late bacterial growth stage (OD_600_ = 2.0) than the early bacterial growth stage (OD_600_ = 0.5), in particular, the expression of *speA*, *speC*, and *potD* were enhanced remarkably along with bacterial proliferation (Fig. 9A). Then we determined the expression levels of genes *speA*, *speD*, *speE*, and *potD* in the mutant Δ*spuE* cultured in MMN medium at an OD_600_ of 1.2. The results showed that expression of *speA*, *speD*, and *potD* were increased approximately1.5- to 2.0-fold in the transporter mutant Δ*spuE* compared with the wild-type strain PAO1, but a significant change in *speE* transcription was not detected in Δ*spuE* compared with wild-type PAO1 (Fig. 9B). These results are agreeable with the findings that spermidine biosynthesis was enhancing along with bacterial growth (Fig. 8A), and that expression of the spermidine synthase gene *speE* was auto-induced by spermidine, which requires a functional spermidine transporter SpuDEFGH (Fig. 8B). It is interesting to note that null mutation of *spuE* led to much increased expression of *speA*, *speD*, and *potD* which encode enzymes and proteins involved in putrescine and spermidine biosynthesis and uptake, suggesting a compensation mechanism for keeping intracellular polyamine molecules at a critical level.

### Interaction of spermidine-related genes with other T3SS regulatory mechanisms.

As discussed in the Introduction section, T3SS gene expression is also modulated by cAMP-Vfr and GacA/S signaling mechanisms. To elucidate the relationship between spermidine signaling pathway and the above two regulatory mechanisms, we determined the mRNA level of *vfr*, *cyaB*, *gacA*, *gacS*, and *ladS* in wild-type strain PAO1, mutants Δ*speE* and Δ*spuE*, and vice versa, the mRNA level of *speA*, *speD*, *speE*, *spuE*, and *potD* in wild-type strain PAO1, mutants Δ*vfr* and Δ*gacA*. The results showed that expression levels of *vfr*, *gacA*, *gacS*, and *ladS* were comparable in the mutants Δ*speE* and Δ*spuE* compared with those in wild-type strain PAO1, but the transcripts of *cyaB*, which encodes cAMP biosynthesis, was increased in mutant Δ*speE* (Fig. S6A and B). Expression levels of *speA*, *speD*, and *potD* were enhanced in mutant Δ*vfr* but decreased in mutant Δ*gacA* (Fig. S6C), while expression levels of *speE* and *spuE* in mutants Δ*vfr* and Δ*gacA* were similar to wild-type strain PAO1 (Fig. S6D). These results suggest the potential interaction or cross-talking among the above-mentioned three signaling regulatory mechanisms.

## DISCUSSION

Our previous study showed that T3SS gene expression in P. aeruginosa could be induced by spermidine and spermine produced by mammalian hosts, and in this process the bacterial membrane ABC transporter SpuDEFGH played a critical role in influx of and responding to host spermidine signals (33). In this study, we investigated the role of bacterial endogenously synthesized spermidine molecules in modulation of T3SS in P. aeruginosa. Given that putrescine is a key substrate for biosynthesis of spermidine (37–39), we deleted the genes involved in putrescine biosynthesis and transportation, along with the genes encoding spermidine synthases and transportation for functional analysis and comparison. The investigation unveiled several interesting new findings. Firstly, we demonstrated that the bacterial endogenously synthesized spermidine is a key and specific signal in modulation of T3SS gene expression and the bacterial virulence. Secondly, we found that the regulatory role of endogenously synthesized spermidine did not totally rely on the spermidine transporter SpuDEFGH but needed the transporter for its full functionality in regulation of T3SS. Thirdly, this study provided evidence that spermidine signaling mechanism could interact or cross talk with cAMP-Vfr and GacS/GacA regulatory mechanisms. In addition, unexpectedly, our data seem to suggest the presence of an alternative spermidine biosynthesis mechanism independent of putrescine.

Reminiscent to the spermidine signal from host organisms that regulates T3SS expression through host-pathogen communication (33), our results showed that endogenously synthesized spermidine molecules could act as a QS signal to modulate T3SS expression in P. aeruginosa. When cultured in MMN medium, deletion of the spermidine synthase gene *speE* led to substantially decreased cellular level of spermidine (Fig. 4C), a significant reduction in T3SS gene expression (Fig. 2B and C), much decreased protein levels of the effector ExoS and T3SS structural protein PcrV (Fig. 7), and attenuated cytotoxicity on human cell line A549 (Fig. 6). Consistent with the above findings, transcriptome analysis showed that a total of 38 T3SS genes were significantly downregulated in the *speE* deletion mutant (Fig. 8C; Table S3), which was validated by RT-qPCR analysis (Fig. 8D). Interestingly, we found the transcriptional expression *speE*, as well as *speD* encoding dSAM synthesis, could be augmented along with bacterial proliferation (Fig. 9A), and *speE* expression was induced by spermidine in a dosage-dependent manner (Fig. 9B). The above findings, together with our previous study on the role of host spermidine signal in modulation of T3SS (33), indicate that P. aeruginosa could respond to the spermidine signals from both host organisms and endogenously produced to activate T3SS during pathogen-host interaction. Such a mechanism may obviously aid the bacterial pathogen to overcome host defense mechanisms in the process of acute infection, as it enables the pathogen to exploit host spermidine signal to stimulate endogenous spermidine biosynthesis and thus boost cellular signal level for upregulation of T3SS, which is a key virulence determinant of P. aeruginosa (6, 11, 15, 33, 34).

To determine whether the bacterial membrane transporters are required for endogenous spermidine signaling in modulation of T3SS, we used two media for analysis and functional comparison, including LBN that contains polyamines (33), and MMN that did not contain polyamine molecules. Null mutation of *speE* and *speD*, which encodes spermidine and dSAM biosynthesis, respectively, led to over 60% decrease in the expression level of *exsCEBA* in MMN medium, and about 30% reduction in LBN medium (Fig. 2B and C). The findings indicate a possibility that the *speE* mutant could uptake spermidine molecule from LBN medium. This notion was supported by the results that deletion of *spuE*, which encodes the substrate binding protein of the spermidine transporter SpuDEFGH, also caused a significant reduction in *exsCEBA* expression. The expression of *exsCEBA* was further reduced in the double deletion mutant Δ*speE*Δ*spuE*, and in particular, the double deletion mutant Δ*speE*Δ*spuE* displayed a similar basal level expression of *exsCEBA* in both LBN and MMN media (Fig. 3B). Similar with this trend, the transcriptional level of *exsCEBA*, effector protein ExoS, secretion protein PcrV, and bacteria cytotoxicity could not be fully rescued with *in trans* expression of *speE* or *spuE* in the double mutant Δ*speE*Δ*spuE* (Fig. 3B, 6, and 7), suggesting that P. aeruginosa inclined to respond and utilize exogenous spermidine when lack of spermidine biosynthase. In contrast, deletion of *potD*, which is a predicted substrate binding protein showing about 32% similarity to the spermidine binding protein of E. coli transporter PotABCD (42), did not lead any significant change in the expression level of *exsCEBA* in comparison with the *spuE* mutant (Fig. 3A). Given that deletion of either *speE* or *spuE* could cause about 50% reduction in bacterial cytotoxicity (Fig. 6), we conclude that spermidine transporter SpuDEFGH is not essential for endogenous spermidine signaling but required for its full functionality in modulation of T3SS in P. aeruginosa. Interestingly, expression levels of the double deletion mutant Δ*speE*Δ*spuE* complemented with the spermidine synthase gene *speE* were higher than that complemented with the transporter gene *spuE* in MMN medium (Fig. 7). These findings are agreeable with the fact that LBN but not MMN medium contains polyamine molecules (33), suggesting that SpuDEGHF transporter plays a dominant role in modulating T3SS gene expression in the environment with spermidine signal and the endogenous spermidine biosynthesis is more important than the SpuDEGHF transporter in regulating T3SS gene expression under the condition without external spermidine resource, further confirming the essential role of spermidine signal in modulation of T3SS. Furthermore, exogenous addition of spermidine could slightly increase the T3SS gene expression level in the transporter mutants Δ*spuE*Δ*potD* and Δ*speE*Δ*spuE* (*P < *0.05) compared with the corresponding control without exogenous spermidine (Fig. 5), which suggests that other transporter(s) may play a minor role in influx of spermidine in P. aeruginosa.

RNA-seq results showed that deletion of spermidine synthase gene *speE* led to a significant reduction in the transcript levels of most if not all the T3SS-related genes in P. aeruginosa (Fig. 8C and D; Table S3). In addition, a few genes encoding transcriptional regulation and metabolism were also downregulated in the absence of spermidine synthase (Table S3), which prompted us to investigate the potential interaction or cross-talking among signaling pathways or regulatory mechanisms. On the top of the central ExsCEBA regulatory cascade, cAMP-Vfr system is known responding to calcium depletion stress (43), GacS/GacA system may be activated by sugar molecules (42), and spermidine signaling is initiated when pathogen interacting with host cells (33). RT-qPCR performed between the wild-type strain PAO1 and corresponding mutants showed that expression level of *cyaB* which encodes cAMP biosynthesis, was increased in mutant Δ*speE* (Fig. S6A), and expression of *speD* which encodes dSAM biosynthesis, was enhanced in mutant Δ*vfr* but decreased in mutant Δ*gacA* (Fig. S6C). Given that mutation of *speD* could substantially change the expression patterns of T3SS genes (Fig. 2B), these results seem to suggest potential interactions between spermidine signaling pathway and GacS/GacA and cAMP-Vfr regulatory mechanisms in regulation of T3SS through SpeD, which may be worthy of further investigations.

Previous studies in E. coli demonstrated that spermidine was synthesized using putrescine and dSAM as substrate (41, 44), and bioinformatics analysis showed that all the genes in this pathway are conserved in P. aeruginosa (Fig. 1 and Fig. S2). However, several lines of evidence from this study seem to rule out the possibility that P. aeruginosa utilize putrescine as a substrate to synthesize spermidine. Firstly, we found that T3SS gene expression in wild-type strain PAO1 was inhibited by exogenous addition of putrescine in a dosage dependent manner from 0.1 to 1 mM (Fig. S4A), and similarly, the T3SS gene expression in other mutants including the deletion mutant of *speA*, which encodes the first enzyme in putrescine biosynthesis pathway (Fig. 1), was also significantly downregulated by exogenously added putrescine (Fig. S4B). On the contrary, exogenous addition of spermidine could substantially increase T3SS gene expression in wild-type strain PAO1 and in the spermidine synthase gene mutant Δ*speE* (Fig. 8). Secondly and critically, spermidine levels in mutants Δ*speE*, Δ*speD*, Δ*spuE*, and Δ*speE*Δ*spuE* were decreased markedly (Fig. 4A to C), but remained unchanged in the mutants Δ*speA*, Δ*speC*, and Δ*speA*Δ*speC* compared with wild-type strain PAO1 in both LBN and MMN media (Fig. 4D to F). Given that deletion of *speD* also resulted in much reduced spermidine biosynthesis (Fig. 4A to C), it seems plausible that P. aeruginosa may utilize dSAM and another amino acid or derivative instead of putrescine to synthesize spermidine. In this regard, it is interesting to note that the SpeE homologue from Helicobacter pylori could not bind and use putrescine and dSAM as substrates to synthesize spermidine (45). Besides, in *Thermus thermophiles*, spermidine is generated directly by SpeB from SpeE-generated N^1^-aminopropylagmatine but not putrescine (46). Bioinformatics analysis showed that SpeB in Thermus thermophilus shares 31.1% similarity with GbuA and GpuA, respectively, which is involved in the metabolism from l-arginine to succinate (ATA pathway) in P. aeruginosa. The role of GbuA and GpuA in the biosynthesis of spermidine in P. aeruginosa deserves further investigation.

In summary, in this study we present evidence that endogenously synthesized spermidine acts as a QS signal, playing a key role in regulation of T3SS gene expression and the virulence of P. aeruginosa, and its full functionality requires the ABC transporter SpuDEFGH. These findings together with the previous report on the role of spermidine in host-pathogen communication (33) strongly indicate that the transporter SpuDEFGH is an important target for design and develop new therapies to control P. aeruginosa infections, as it could tap to both host and bacterial cells released spermidine signal to activate and boost the bacterial T3SS gene expression. In addition, the results from this study also present several intriguing clues and puzzles, for example, the roles of putrescine, which seems under the control of GacS/GacA system (Fig. S6C and D), in P. aeruginosa physiology and T3SS regulation, spermidine biosynthesis pathway in P. aeruginosa, and the detailed molecular mechanisms of spermidine signal in modulation of T3SS, which remain to be investigated.

## MATERIALS AND METHODS

### Strains and culture conditions.

The bacterial strains and mutants used in this study were listed in Table S1. P. aeruginosa PAO1 was used as a parental strain for generation of reporter strains and deletion mutants. The primers for gene cloning and knockout, PCR and RT-qPCR analysis were listed in Table S2. Unless otherwise indicated, bacterial cells were routinely grown at 37°C in Luria-Bertani broth (LB) or minimal medium containing trisodium nitrilotriacetic acid (MMN, 25 mM KH_2_O_4_, 95 mM NH_4_Cl, 50 mM monosodium glutamate, 110 mM disodium succinate, 10 mM trisodium nitrilotriacetic acid, 2.5% glycerol, 5 mM MgSO_4_, and 18 μM FeSO_4_) (47). Antibiotics were added to medium when necessary: gentamicin (Gm), 50 μg/mL for P. aeruginosa and E. coli; kanamycin (Km), 50 μg/mL for E. coli; ampicillin, 100 μg/mL for E. coli; tetracycline (Tc), 150 μg/mL for P. aeruginosa and 15 μg/mL for E. coli. Chelating reagent nitrilotriacetic acid (NTA) at a final concentration of 7.5 mM was added into LB medium for induction of T3SS expression. For generation of in-frame deletion mutants, the gene replacement vector pK18mobsacB derivatives ligated with the 500 bp upstream and 500 bp downstream of target genes were transformed into corresponding parental strains with the helper vector pRK2013 by triparental mating. For the construction of complementation and overexpression strains, the promoter and ORF region of target genes were cloned into the digested pBBR1-MCS5, transformed into corresponding strains by triparental mating. All the resultant constructs were confirmed by PCR analysis and DNA sequencing.

### RNA extraction and quantitative real-time PCR.

Total RNA samples were isolated from fresh bacterial cultures using Ribopure bacterial RNA isolation kit following the instructions from the manufacturer (Ambion Inc., USA) and digested with DNase I (Invitrogen) to remove residue genomic DNA. Quantity and purity of RNA samples were determined by agarose gel electrophoresis and spectrometry analysis. The cDNA samples were synthesized from total RNA samples by using TransScript First-Strand cDNA Synthesis kit following the protocol of manufacturer (TransGen Biotech, China). Quantitative real-time PCR (RT-qPCR) was performed by using PowerUp SYBR green master mix and QuantStudio 6 Flex real-time PCR system (Thermo Fisher Scientific) in standard cycling mode. The transcript level of *rpoD* gene was set as the reference for data analysis.

### Transcriptome analysis.

Total RNA of each sample was extracted using TRIzol Reagent or RNeasy minikit (Qiagen). Total RNA of each sample was quantified and qualified by Agilent 2100/2200 Bioanalyzer (Agilent Technologies, Palo Alto, CA, USA), NanoDrop (Thermo Fisher Scientific Inc.), and 1% agarose gel. An aliquot of 1 μg total RNA was used for library preparation. Next generation sequencing library preparations were constructed according to the manufacturer’s protocol. The rRNA was depleted from total RNA using rRNA removal Kit. The ribosomal depleted RNA was then fragmented and reverse-transcribed. First strand cDNA was synthesized using ProtoScript II Reverse Transcriptase with random primers and Actinomycin D. The second-strand cDNA was synthesized using Second Strand Synthesis Enzyme Mix (include dACGTP/dUTP). The purified double-stranded cDNA by beads was then treated with End Prep Enzyme Mix to repair both ends and add a dA-tailing in one reaction, followed by a T-A ligation to add adaptors to both ends. Size selection of adaptor-ligated DNA was then performed using beads, and fragments about 400 bp (with the approximate insert size of 300 bp) were recovered. The dUTP-marked second strand was digested with Uracil-Specific Excision Reagent enzyme. Each sample was then amplified by PCR using P5 and P7 primers, with both primers carrying sequences which can anneal with flow cell to perform bridge PCR and P5/P7 primer carrying index allowing for multiplexing. The PCR products were cleaned up using beads, validated using an Qsep100 (Bioptic, Taiwan, China), and quantified by Qubit3.0 Fluorometer (Invitrogen, Carlsbad, CA, USA). Then libraries with different indices were multiplexed and loaded on an Illumina HiSeq/Novaseq instrument according to manufacturer’s instructions (Illumina, San Diego, CA, USA) or a MGI2000 instrument according to manufacturer’s instructions (MGI, Shenzhen, China). Sequencing was carried out using a 2 × 150 paired-end (PE) configuration; image analysis and base calling were conducted by the HiSeq Control Software (HCS) + OLB + GAPipeline-1.6 (Illumina) on the HiSeq instrument.

### Quantitative *β*-galactosidase assay.

Overnight bacterial cultures were diluted 1:100 in fresh LB medium supplemented with NTA, MMN, or MMN containing spermidine as indicated. Bacterial cell cultures were shaking at 37°C until the OD_600_ reaching about 1.3. The *β*-Galactosidase activity was measured as previously described (48). Results were the averages from at least three independent experiments and given as Miller units (MU).

### Protein isolation and Western blot assay.

Overnight bacterial cultures were diluted 1:1,000 in the fresh LB medium supplemented with NTA or MMN medium. The bacterial cells were grown at 37°C till OD_600_ reaching about 1.3, which were then chilled on ice for 10 min and collected by centrifugation. The supernatants and bacterial pellets were used for preparation of extracellular proteins and total cellular proteins, respectively. For isolation of total cellular proteins, the bacterial pellets were resuspended in PBS buffer supplemented with protease inhibitor and the cells were broken by sonification and then centrifuged to collect protein solutions. The supernatants from bacterial cultures were filtered with 0.2 μm syringe filter and precipitated with trichloroacetic acid (TCA) at a final concentration of 10% (vol/vol) or centrifuged with ultrafiltration tubes. The precipitates were washed with acetone, dried, and resuspended in SDS buffer. The protein samples were denatured by boiling for about 5 min and separated by SDS-PAGE. Western blot analysis was performed following the standard protocols.

### A549 cell culture and cytotoxicity assay.

Bacterial cytotoxicity was determined using human lung epithelial cell A549. A549 cells were seeded in 96-well tissue culture plates with Dulbecco’s Modified Eagle Medium (DMEM) containing 10% bovine serum and allowed to grow at 37°C with 5% CO_2_ for overnight. The cell culture supernatants were removed, the monolayer was washed once with PBS buffer. Overnight bacterial cultures were subcultured in fresh LB and cultured till exponential growth phase before infection. Bacterial cells were centrifuged and resuspended in DEME containing 1% bovine serum. A549 cells were infected with the bacterial cells at a multiplicity of infection (MOI) of 50. Bacterial cytotoxicity was determined by measuring the activity of released lactate dehydrogenase (LDH) in the culture supernatants by using the cytotoxicity detection kit (Promega) at indicated time points postinfection.

### Spermidine extraction, derivatization, and detection.

Cell lysates were collected as described above. Derivatization was executed by adding 100 μL benzoyl chloride and 1 mL of 2 M NaOH to same volume of cell lysate sample, vortexed for 20 s, and then the mixture was incubated for 20 min at 37°C. After that, 2 mL saturated NaCl solution was added and vortexed for 20 s prior to the addition of petroleum ether for extracting derivatized polyamines. Benzoylated spermidine was detected by Thermo Fisher Q Exactive Focus high performance liquid chromatography mass spectrometry (LC-MS).

### Accession of transcriptome data.

The raw data of transcriptome is accessible under SRA accession number PRJNA808670 in NCBI.
