# Supplementary material for: Spermidine Is an Intercellular Signal Modulating T3SS Expression in Pseudomonas aeruginosa
Source: Microbiol Spectr. 2022 Apr 18;10(3):e00644-22. doi: 10.1128/spectrum.00644-22 (PMC9241758; doi:10.1128/spectrum.00644-22)
Supplement: SUPPLEMENTAL FILE 1 — Supplemental material. Download spectrum.00644-22-s001.pdf, PDF file, 1.3 MB [file spectrum.00644-22-s001.pdf]

Supplementary Information

Spermidine is an intercellular signal modulating T3SS expression in *Pseudomonas aeruginosa*

Content:

Supplementary Table S1. Bacterial strains and plasmids used in this study.

Supplementary Table S2. Primers used in this study

Supplementary Table S3. Genes regulated by SpeE

Supplementary Figure S1. Bacterial growth of *Pseudomonas aeruginosa* PAO1 and its derivatives in different media

Supplementary Figure S2. Sequence alignment of SpuE and PotD from *Pseudomonas aeruginosa* PAO1with the spermidine binding protein PotD from *Escherichia coli*

Supplementary Figure S3. Transcriptional expression of *exsCEBA* in *Pseudomonas aeruginosa* PAO1 and its derivatives in MMN medium

Supplementary Figure S4. Transcriptional expression of *exsCEBA* in *Pseudomonas aeruginosa* PAO1 and its derivatives with or without putrescine

Supplementary Figure S5. Gene ontology analysis of differentially expressed genes between *Pseudomonas aeruginosa* PAO1 and mutant Δ*speE*

Supplementary Figure S6. Interaction of spermidine-related genes with other T3SS regulatory mechanisms in *Pseudomonas aeruginosa* PAO1

Supplementary Figure S7. Full images of the Western blots used in this study

Table S1. Bacterial strains and plasmids used in this study

Strains or plasmids	Relevant description*	Source or reference
**Bacterial strains**		
Escherichia coli		
DH5α	F-φ80d *lacZ*ΔM15 Δ (*lacZ*YA-*arg*F) U169 end A1 recA1 *hsd*R17($r_k^-m_k^+$) *sup*E44λ- thi-1 *gyr*A96 *rel*A1 *pho*A for plasmid transformation	Lab collection
pRK2013	*Thr leu thi recA hsdR hsdM pro*, Km^r	Lab collection
Pseudomonas aeruginosa		
PAO1	Prototrophic laboratory strain	Lab collection
Δ*speA*	*speA* in-frame deletion mutant from PAO1	This study
Δ*speC*	*speC* in-frame deletion mutant from PAO1	This study
Δ*speD*	*speD* in-frame deletion mutant from PAO1	This study
Δ*speE*	*speE* in-frame deletion mutant from PAO1	This study
Δ*spuE*	*spuE* in-frame deletion mutant from PAO1	This study
Δ*potD*	*potD* in-frame deletion mutant from PAO1	This study
Δ*aguA*	*aguA* in-frame deletion mutant from PAO1	This study
Δ*aguB*	*aguB* in-frame deletion mutant from PAO1	This study
Δ*speA*Δ*potD*	*speA* and *potD* in-frame deletion mutant from PAO1	This study
Δ*speD*Δ*potD*	*speA* and *potD* in-frame deletion mutant from PAO1	This study
Δ*spuE*Δ*potD*	*spuE* and *potD* in-frame deletion mutant from PAO1	This study

Δ*speE*Δ*spuE*	*speE* and *spuE* in-frame deletion mutant from PAO1	This study
Δ*vfr*	*vfr* in-frame deletion mutant from PAO1	This study
Δ*gacA*	*gacA* in-frame deletion mutant from PAO1	This study
PAO1pClacZ	*lacZ* fused to the *exsCEBA* promoter and integrated at the *attB* site of the PAO1 chromosome	(1)
Δ*speA*pClacZ	*speA* in-frame deletion mutant from PAO1pClacZ	This study
Δ*speC*pClacZ	*speC* in-frame deletion mutant from PAO1pClacZ	This study
Δ*speD*pClacZ	*speD* in-frame deletion mutant from PAO1pClacZ	This study
Δ*speE*pClacZ	*speE* in-frame deletion mutant from PAO1pClacZ	This study
Δ*spuE*pClacZ	*spuE* in-frame deletion mutant from PAO1pClacZ	This study
Δ*potD*pClacZ	*potD* in-frame deletion mutant from PAO1pClacZ	This study
Δ*aguA*pClacZ	*aguA* in-frame deletion mutant from PAO1pClacZ	This study
Δ*aguB*pClacZ	aguB in-frame deletion mutant from PAO1pClacZ	This study
Δ*speA*Δ*potD*pClacZ	*speA* and *potD* in-frame deletion mutant from PAO1pClacZ	This study
Δ*speD*Δ*potD*pClacZ	*speA* and *potD* in-frame deletion mutant from PAO1pClacZ	This study
Δ*spuE*Δ*potD*pClacZ	*spuE* and *potD* in-frame deletion mutant from PAO1pClacZ	This study
Δ*speE*Δ*spuE*pClacZ	*speE* and *spuE* in-frame deletion mutant from	This study

	PAO1pClacZ	
**Plasmids**		
pBBR1-MCS5	Broad-host-range expression vector, Gm^r	Lab collection
pBBR1-*speD*	*speD* encoding region cloned in pBBR1-MCS5, Gm^r	This study
pBBR1-*speE*	*speE* encoding region cloned in pBBR1-MCS5, Gm^r	This study
pBBR1-*spuE*	*spuE* encoding region cloned in pBBR1-MCS5, Gm^r	This study
pBBR1-*potD*	*potD* encoding region cloned in pBBR1-MCS5, Gm^r	This study

*: Gm^r, gentamicin resistance; Kan^r, kanamycin resistance.

REFERENCE

1. Zhou L, Wang J, Zhang LH. 2007. Modulation of bacterial type III secretion system by a spermidine transporter dependent signaling pathway. PLoS One 2:e1291.

Table S2. Primers used in this study

Primer	Sequence (5’-3’)
**For in-frame deletion**	
speA-up-F	gagctcggtacccggggatccCGCCTGTCGGCGACGGGA
speA-up-R	agGCGCGGTGATCTCCTCAAT
speA-down-F	ttgaggagatcaccgcgcCTGGCTAGCCCGTCGGCG
speA-down-R	acgacggccagtgccaagcttGGTCGCGGCAAGGCCCTG
speC-up-F	gagctcggtacccggggatccCGCCCAGGTGACCCAGCT
speC-up-R	tgcgGGTTGGGACTCCCAATGGC
speC-down-F	attgggagtcccaaccCGCAGTCGCCTCTGCTACG
speC-down-R	acgacggccagtgccaagcttACGGGTTGTAGGCAATTTCCC
speD-up-F	CGGGATCCGCCCAAGGTGTTCACGAAGATCC
speD-up-R	TTCTTCCTCGTGTGCGACGTGGGGAACTCTCTCGG
speD-down-F	CCGAGAGAGTTCCCCACGTCGCACACGAGGAAGAA
speD-down-R	CCCAAGCTTAGGCGCTGTACCAGGGCCA
speE-up-F	gagctcggtacccggggatccCCGGATTGGCGGCCACCA
speE-up-R	ttcttggtgaagcgGGGCCGGGATCTCCCTGG
speE-down-F	cggcccCGCTTCACCAAGAAGGGCC
speE-down-R	acgacggccagtgccaagcttCCTGCTCCTGGCTGATCGC
aguA-up-F	gagctcggtacccggggatccGGACCAGTGGTTCCCCGA

aguA-up-R	gCGCTGCTCCTGAAATCGTTG
aguA-down-F	aacgatttcaggagcagcgCGGGCATCCCGCAGGGCG
aguA-down-R	acgacggccagtgccaagcttGATGGCGAAGGTTCTACCGC
aguB-up-F	gagctcggtacccggggatccCGGCGGCCCGCCAACTGA
aguB-up-R	atcgtttcccgaggccGGAGCACCTCGTGTGCTGG
aguB-down-F	ctccGGCCTCGGGAAACGATCC
aguB-down-R	acgacggccagtgccaagcttTCTTGCGTGCCACCTGGT
spuE-up-F	CGGGATCCCTGTATACCTTCCCCGACCTGCC
spuE-up-R	ACCGGGCGGAATTTGAACGTCCGACTCCTGTTCCGG
spuE-down-F	CCGGAACAGGAGTCGGACGTTCAAATTCCGCCCGGT
spuE-down-R	CCCAAGCTTTTGCGCTTGGCGTACTGGG
potD-up-F	CGGGATCCCATGAGCCTGGGCATGCTCTC
potD-up-R	CGTCGATAGCGGTGCGGAGGTTCCTTGTTACGGGGT
potD-down-F	ACCCCGTAACAAGGAACCTCCGCACCGCTATCGACG
potD-down-R	CCCAAGCTTCGAGCAACTGGCGATAGCCG
vfr-up-F	gagctcggtacccggggatccCGGCCTCGAGGAAGGCTT
vfr-up-R	tttttcatgggtgctgtGCCCGAGTCCCGAAAGAA
vfr-down-F	ggcACAGCACCCATGAAAAAGGCC
vfr-down-R	acgacggccagtgccaagcttCGAGGCGCGGGCGATCGG
gacA-up-F	gagctcggtacccggggatccCATCAGGAAGCAATCCTGGATC

gacA-up-R	aaaacggcgctcatGCTGCACCTCGTCGCGCA
gacA-down-F	tgcagcATGAGCGCCGTTTTCGACG
gacA-down-R	acgacggccagtgccaagcttCAGGGCCGCGTACGGTTG
**For in *trans* complementation**	
pBBR1-*speD*-F	gtcgacggtatcgataagcttAAAAGGGGCGCAAACTCTG
pBBR1-*speD*-R	cgctctagaactagtggatccTCAGCGCGGCATGTTGCG
pBBR1-*speE*-F	gtcgacggtatcgataagcttCCGTTGGCCCGGAAGCTG
pBBR1-*speE*-R	cgctctagaactagtggatccTCAGTCGTTGTCCTGCTTGCC
pBBR1-*spuE*-F	gtcgacggtatcgataagcttTCGTCCAGACGCTTCCTCC
pBBR1-*spuE*-R	cgctctagaactagtggatccTTATTTGCCGGTCTTGATCCG
pBBR1-*potD*-F	gtcgacggtatcgataagcttTGATGGCCTTCACCCTGTCC
pBBR1-*potD*-R	cgctctagaactagtggatccTCAGCGCAGCTTGATCTCTGT
**For RT-qPCR**	
rpoD-F	GCGAAGAAGGAAATGGTC
rpoD-R	CGTAGGTGGAGAACTTGTA
speA-F	CAGTGCTACTTCGCTATCT
speA-R	CTTGTCGTTGAGTTCGTC
speC-F	TGGACAAGGTGATGAAGA
speC-R	TCGTAGAAGTAGCGGATG
aguA-F	TGAACTTCCTGATCGTCAA

aguA-R	GGTGATGCAATGGATGTT
aguB-F	CTATGGTTCTTCGTTCATCG
aguB-R	AGGTGTGGACAAGGATAC
speD-F	ATCTACGACATCTGCTACG
speD-R	GACAACATCGGTAAGAATCTG
speE-F	AGCGATTATCAGGAAACTCT
speE-R	TCTCGTGGTAGATGAACTC
potD-F	CGTCGAGCTATATCAAGGA
potD-R	TGTGGATCACCATGTTGT
spuE-F	CAACATTCCATTGGCAAGA
spuE-R	TGTAATCGGTCCAGTTATAGAT
cyaB-F	TGTTCTTCTCCGACATCC
cyaB-R	CGTTGAGGTAGTTGTTGAG
vfr-F	TATTACCCACACACCCAAA
vfr-R	GTTGAGGTAGCCGATGAT
gacA-F	ATCAAGGTCGTGGTAGTC
gacA-R	ATCTGCGGGCTGATATAG
gacS-F	GACTACCTCACGACCATC
gacS-R	CAGTTCCAGTTGCTTCTC
ladS-F	TGGTGATGCTGATCTACAA

ladS-R	AACGAAGCGATATAGAGGAT
exsA-F	CTGCCCAGTTTCTACAAG
exsA-R	CACAAGCAATTCCTTCAAC
exsC-F	CCGTTTCGATCTGCATTTC
exsC-R	GAAGCATTCGAGGGTCAG
pscD-F	GTGCCCTATGTAGTGCTC
pscD-R	CCCTTTGAGATTGATGATGAAA
popN-F	GGACATCCTCCAGAGTTC
popN-R	CGAAACGGAAACGTAGTG
pcrR-F	CTGATTCCCTGGTTTCTC
pcrR-R	CAACATCACGATCCAGAC
pscN-F	GAGTTGCTGCTGAAGATC
pscN-R	GTAATCGCTGGTTTCGTG
pscP-F	CAGTTGCAGATCGAGATC
pscP-R	CTGGTTGAAGGTGAGTTG

Table S3. Genes regulated by SpeE

Gene category and locus	Gene	Function	Log$_2$ Fold change*	P-value
**Type III secretion system**				
PA0044	*exoT*	exoenzyme T	-4.0407	1.61E-40
PA1690	*pscU*	translocation protein in type III secretion	-1.84564	8.22E-08
PA1691	*pscT*	translocation protein in type III secretion	-1.6881	3.15E-06
PA1692	*pscS*	translocation protein in type III secretion	-1.1615	0.00127
PA1693	*pscR*	type III secretion system protein	-2.48639	8.11E-21
PA1694	*pscQ*	type III secretion system protein	-4.83682	3.15E-80
PA1695	*pscP*	translocation protein in type III secretion	-3.9592	6.58E-65
PA1696	*pscO*	translocation protein in type III secretion	-1.51427	3.08E-05

PA1697	*pscN*	type III secretion system ATPase	-2.82133	1.33E-26
PA1698	*popN*	type III secretion outer membrane protein PopN	-3.50638	8.17E-38
PA1699	*pcr1*		-3.48399	6.14E-29
PA1700	*pcr2*		-1.79336	7.93E-07
PA1701	*pcr3*		-2.87811	1.45E-15
PA1703	*pcrD*	type III secretory apparatus protein PcrD	-3.36648	8.13E-37
PA1704	*pcrR*	transcriptional regulator PcrR	-1.69947	2.88E-06
PA1705	*pcrG*	type III secretion regulator	-1.79839	5.07E-07
PA1706	*pcrV*	type III secretion protein PcrV	-4.21799	1.72E-53
PA1707	*pcrH*	regulatory protein PcrH	-4.43825	1.72E-66
PA1708	*popB*	translocator protein PopB	-4.40396	1.28E-43
PA1709	*popD*	translocator outer membrane protein PopD	-4.69046	6.53E-58
PA1710	*exsC*	exoenzyme S synthesis protein ExsC	-2.77605	1.02E-28

PA1711	*exsE*	hypothetical protein	-1.23985	0.000637
PA1712	*exsB*	exoenzyme S synthesis protein ExsB	-3.05927	3.20E-33
PA1713	*exsA*	exoenzyme S transcriptional regulator ExsA	-2.95927	3.18E-45
PA1714	*exsD*	hypothetical protein	-2.69083	5.99E-34
PA1715	*pscB*	type III export apparatus protein	-2.86764	2.59E-19
PA1716	*pscC*	type III secretion outer membrane protein PscC	-2.96911	2.09E-43
PA1717	*pscD*	type III export protein PscD	-2.67178	5.81E-24
PA1718	*pscE*	type III export protein PscE	-2.06817	1.07E-08
PA1719	*pscF*	type III export protein PscF	-2.09228	1.42E-12
PA1721	*pscH*	type III export protein PscH	-3.28022	1.82E-34
PA1722	*pscI*	type III export protein PscI	-2.69414	1.20E-17
PA1723	*pscJ*	type III export protein PscJ	-3.0222	1.35E-38

PA1724	*pscK*	type III export protein PscK	-1.78256	5.53E-07
PA1725	*pscL*	type III secretion system protein	-2.38542	3.41E-18
PA2191	*exoY*	adenylate cyclase	-4.18803	1.37E-60
PA3841	*exoS*	exoenzyme S	-4.30902	6.72E-47
PA3842	*spcS*	chaperone	-3.71998	7.50E-36
**Type VI secretion system**				
PA2266	*hcpB*	secreted protein Hcp	1.002003	0.000627
**Flagellar synthesis, motility, and chemotaxis**				
PA1100	*fliE*	flagellar hook-basal body complex protein FliE	1.085368	4.45E-05
PA0175	*PA0175*	chemotaxis protein methyltransferase	-1.08066	0.000668
**Two-component system**				
PA0178	*PA0178*	two-component sensor	-1.06677	0.00012
**Transporter**				
PA0913	*mgtE*	Mg transporter MgtE	1.004179	6.44E-06

PA2339	*PA2339*	binding-protein-dependent maltose/mannitol transport protein	-1.12311	0.000549
**Metabolic pathway**				
PA0654	*speD*	S-adenosylmethionine decarboxylase	1.282675	1.57E-07
PA2165	*PA2165*	glycogen synthase	-1.18938	3.14E-06
PA2265	*PA2265*	gluconate dehydrogenase	-1.09456	1.09E-05
PA2323	*PA2323*	glyceraldehyde-3-phosphate dehydrogenase	-1.28194	4.34E-05
PA2378	*PA2378*	aldehyde dehydrogenase	-1.41253	2.94E-10
PA2887	*atuB*	citronellol catabolism dehydrogenase	-1.32205	4.84E-05
PA3459	*PA3459*	glutamine amidotransferase	-1.33493	1.34E-08
PA3460	*PA3460*	acetyltransferase	-1.07438	2.44E-06
PA5297	*poxB*	pyruvate dehydrogenase	-1.04373	3.37E-06
**Ribosome**				
PA4567	*rpmA*	50S ribosomal protein L27	1.070204	0.000578
PA4932	*rplI*	50S ribosomal protein L9	1.024404	0.000979
**Oxidative phosphorylation**				

PA1173	*napB*	cytochrome C protein NapB	-1.21736	0.000551
PA2266	*PA2266*	cytochrome C	-1.1583	3.27E-07
PA3621	*fdxA*	ferredoxin I	1.095163	2.32E-07
**Others**				
PA0617	*PA0617*	bacteriophage protein	1.049665	2.74E-05
PA1985	*pqqA*	coenzyme PQQ synthesis protein A	-1.32691	0.000198
PA2113	*opdO*	pyroglutatmate porin OpdO	-1.19634	1.61E-06
PA4138	*tyrS*	tyrosine--tRNA ligase	1.46434	1.01E-07
**Hypothetical protein**				
PA0126	*PA0126*	hypothetical protein	1.07916	1.11E-05
PA0170	*PA0170*	hypothetical protein	1.477056	1.04E-05
PA0645	*PA0645*	hypothetical protein	1.030982	0.00055
PA0788	*PA0788*	hypothetical protein	1.283784	5.25E-05
PA1791	*PA1791*	hypothetical protein	1.066776	6.66E-06
PA2109	*PA2109*	hypothetical protein	-1.53548	2.25E-08
PA2110	*PA2110*	hypothetical protein	-1.18739	3.95E-05
PA2111	*PA2111*	hypothetical protein	-1.82219	5.38E-15

PA2112	*PA2112*	hypothetical protein	-1.27843	8.55E-07
PA2312	*PA2312*	hypothetical protein	-1.33468	0.00011
PA2459	*PA2459*	hypothetical protein	1.049656	0.000162
PA2708	*PA2708*	hypothetical protein	-1.19019	6.13E-05
PA3282	*PA3282*	hypothetical protein	-1.0454	0.000322
PA3785	*PA3785*	hypothetical protein	-1.04675	3.18E-06
PA3939	*PA3939*	hypothetical protein	1.024211	0.000547
PA4013	*PA4013*	hypothetical protein	1.01834	0.001185
PA4507	*PA4507*	hypothetical protein	1.195463	0.00038
PA4580	*PA4580*	hypothetical protein	1.040726	8.27E-05

*Fold changes of gene expression were calculated compared with those in the wild-type strain PAO1.

Supplementary Figure S1

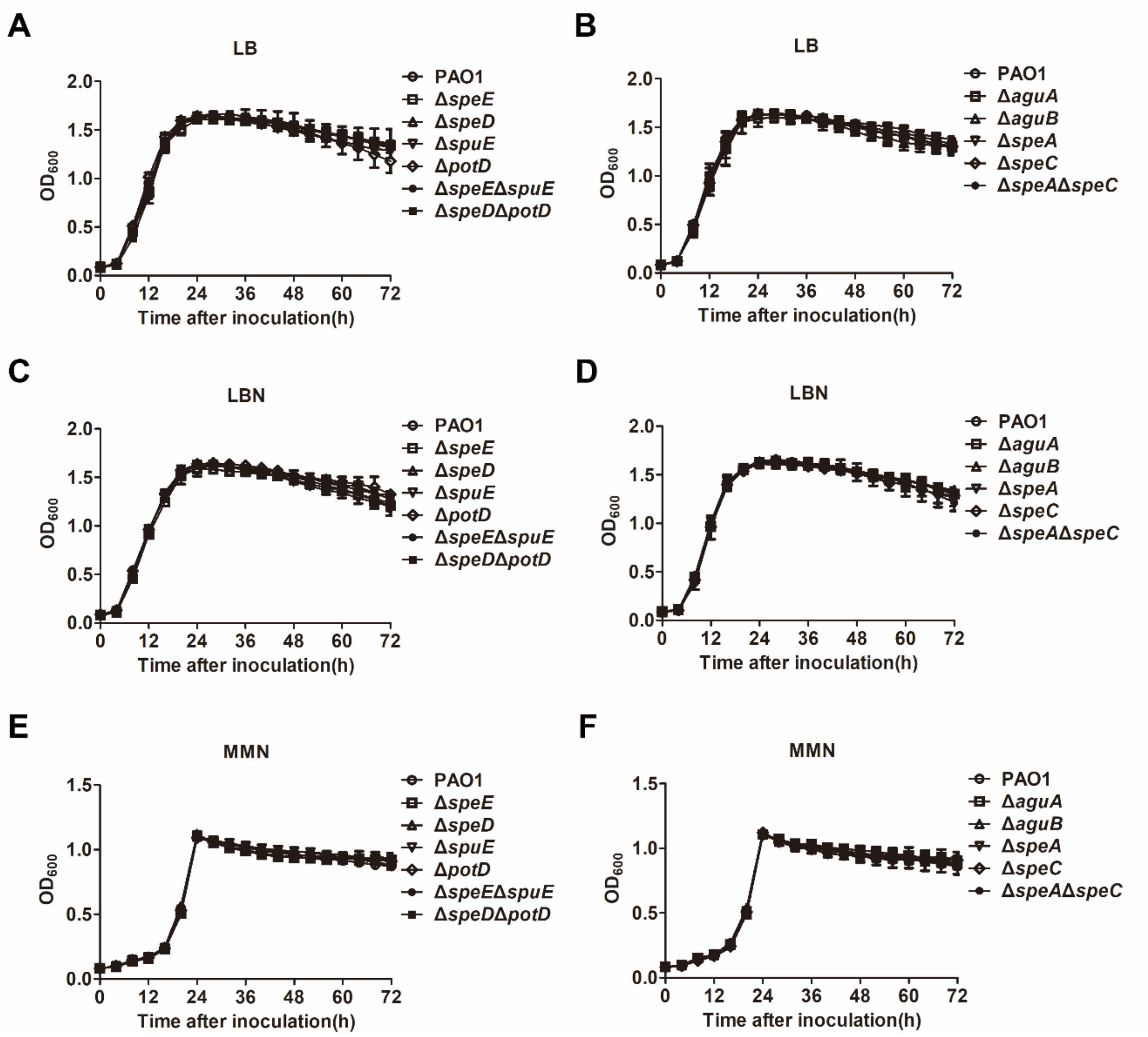

Supplementary Figure S1. Bacterial growth of *Pseudomonas aeruginosa* PAO1 and its derivatives in different media. The wild-type strain PAO1 and its derivatives were cultured in LB (A and B), LBN (C and D), and MMN (E and F) as indicated. Each assay was performed at least triplicates and error bars indicate standard deviations.

Supplementary Figure S2

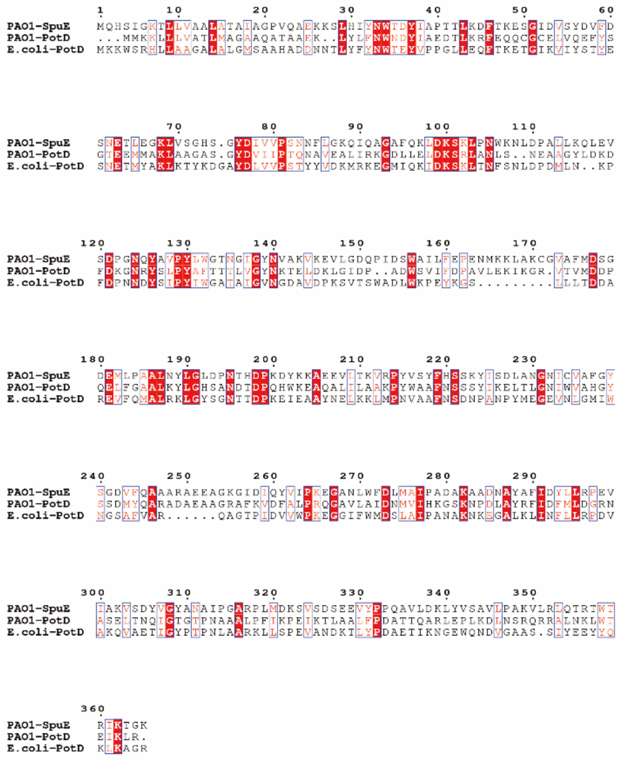

Supplementary Figure S2. Sequence alignment of SpuE and PotD from *Pseudomonas aeruginosa* PAO1 compared with the spermidine binding protein PotD in *Escherichia coli*.

Supplementary Figure S3

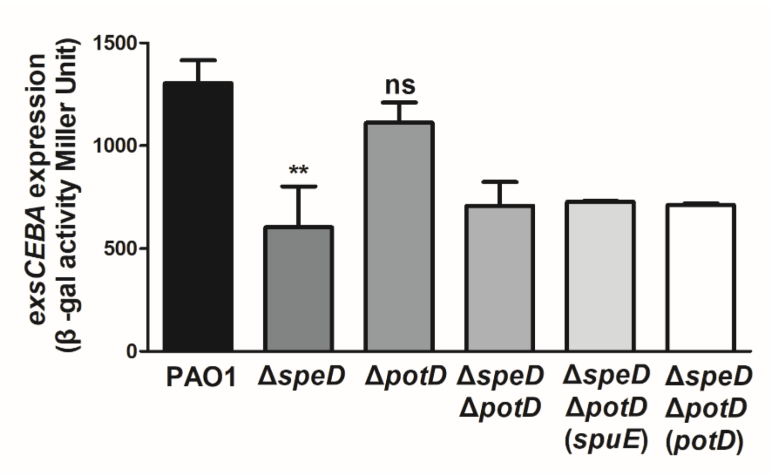

Supplementary Figure S3. Transcriptional expression of *exsCEBA* in *Pseudomonas aeruginosa* PAO1 and its derivatives in MMN medium. The expression of *exsCEBA* was determined in Δ*speD*Δ*potD* with in *trans* expression of *spuE* or *potD*, respectively. Each assay was in at least triplicates and error bars indicate standard deviations. Statistics significance: ns, no significance, **, $P < 0.01$. Statistical analysis compared to wild-type strain PAO1 was performed by using Student's *t* test.

Supplementary Figure S4

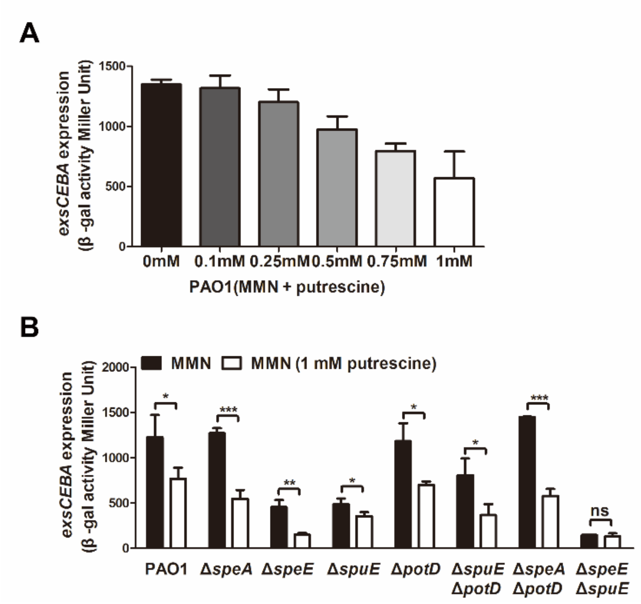

Supplementary Figure S4 Transcriptional expression of *exsCEBA* in *Pseudomonas aeruginosa* PAO1 and its derivatives with or without putrescine. (A) Dosage effect of putrescine on expression of T3SS *exsCEBA* genes in *P*. *aeruginosa* PAO1 cultured in MMN medium. (B) Effect of 1 mM putrescine on expression of *exsCEBA* in *P*. *aeruginosa* PAO1 and derivatives. Each assay was performed at least triplicates and error bars indicate standard deviations. Statistics significance: ns, no significance, *, $P < 0.05$; **, $P < 0.01$; ***, $P < 0.001$. Statistical comparison of strains in MMN and MMN (1 mM putrescine) was performed by using Student's *t* test.

Supplementary Figure S5

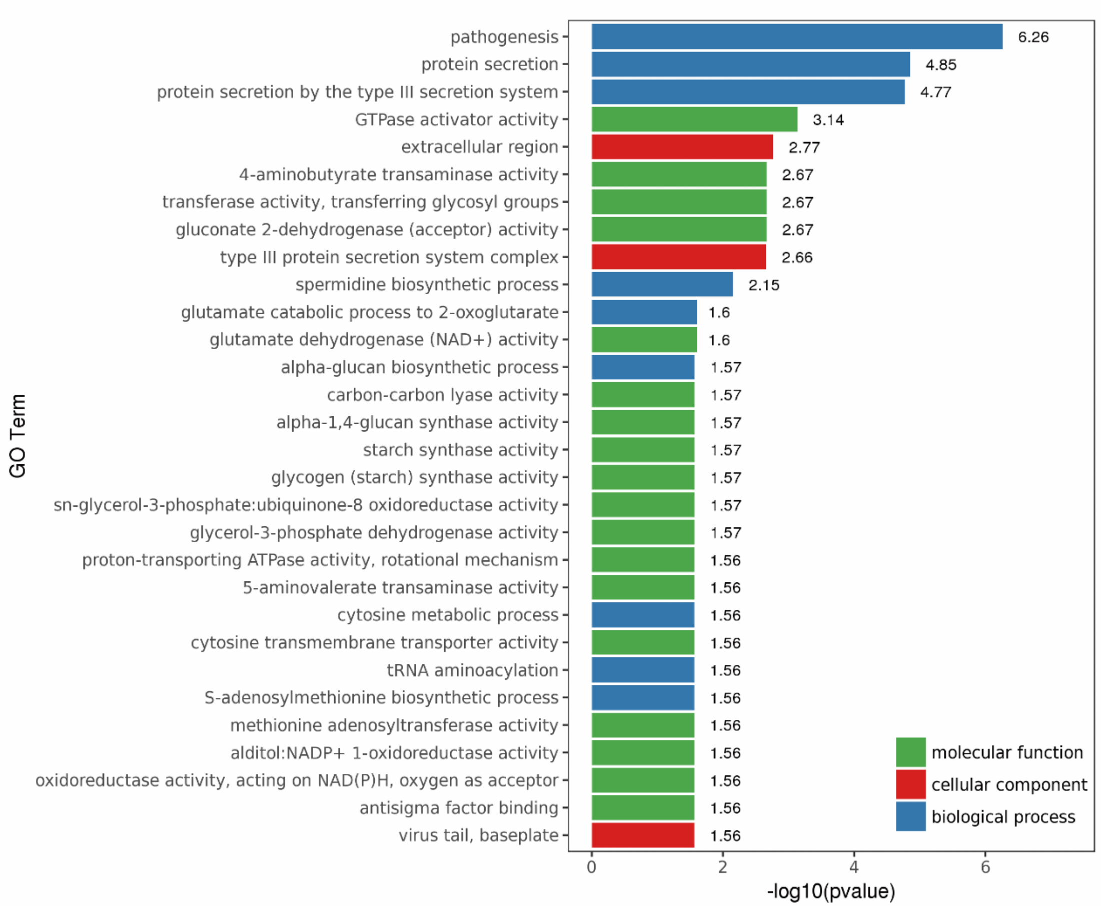

Supplementary Figure S5. Gene ontology analysis of differentially expressed genes between *Pseudomonas aeruginosa* PAO1 and mutant Δ*speE*.

Supplementary Figure S6

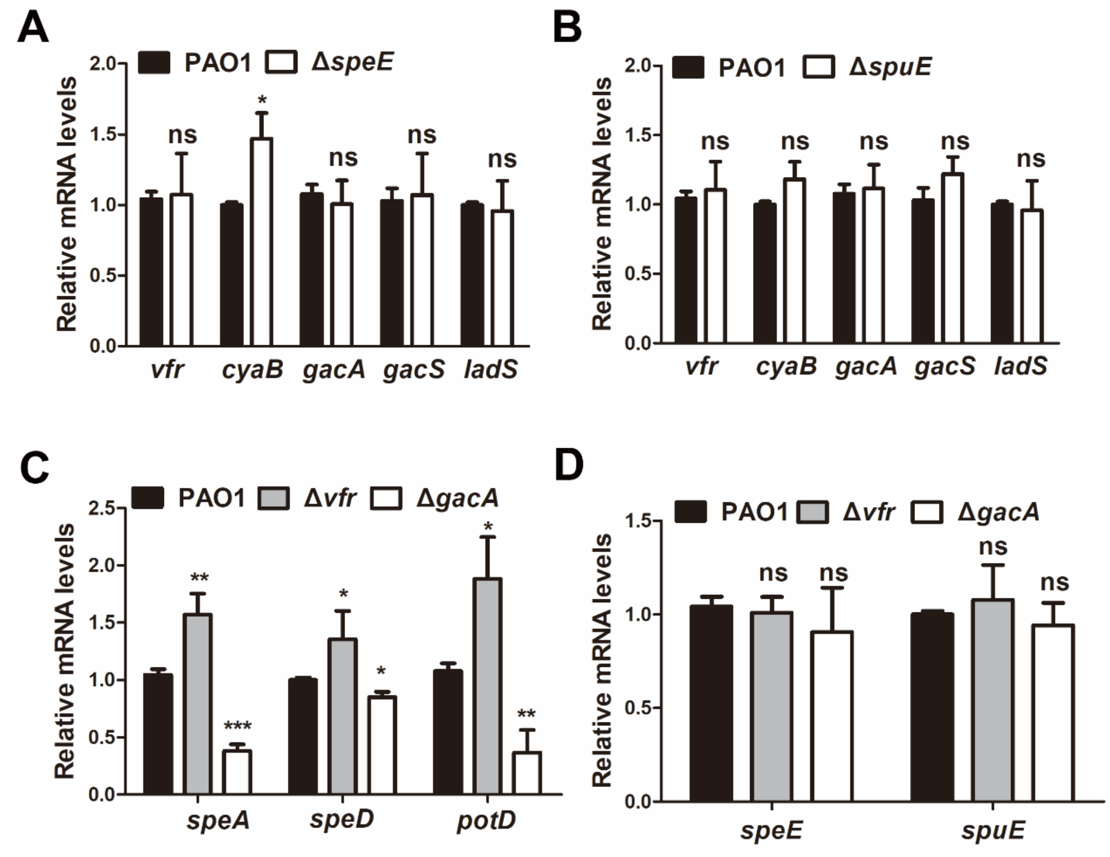

Supplementary Figure S6 Interaction of spermidine-related genes with other T3SS regulatory mechanisms in *Pseudomonas aeruginosa* PAO1. (A) RT-qPCR analysis of the expression levels of *vfr*, *cyaB*, *gacA*, *gacS*, and *ladS* in wild-type strain PAO1 and mutant Δ*speE*. (B) RT-qPCR analysis of the expression levels of *vfr*, *cyaB*, *gacA*, *gacS*, and *ladS* in wild-type strain PAO1 and mutant Δ*spuE*. (C) RT-qPCR analysis of the expression levels of *speA*, *speD*, and *potD* in wild-type strain PAO1, Δ*vfr*, and Δ*gacA* mutants. (D) RT-qPCR analysis of the expression levels of *speE* and *spuE* in wild-type strain PAO1, Δ*vfr*, and Δ*gacA* mutants. Total RNAs were collected when bacteria were grown to an OD_{600} of 1.2. The *rpoD* gene was used as an internal control. The experiment was repeated

at least triplicates in each assay. The error bars indicate standard deviations. Statistics significance: ns, no significance, *, $P < 0.05$; **, $P < 0.01$; ***, $P < 0.001$. Statistical comparison to wild-type strain PAO1 was performed by using Student's t test.

Supplementary Figure S7

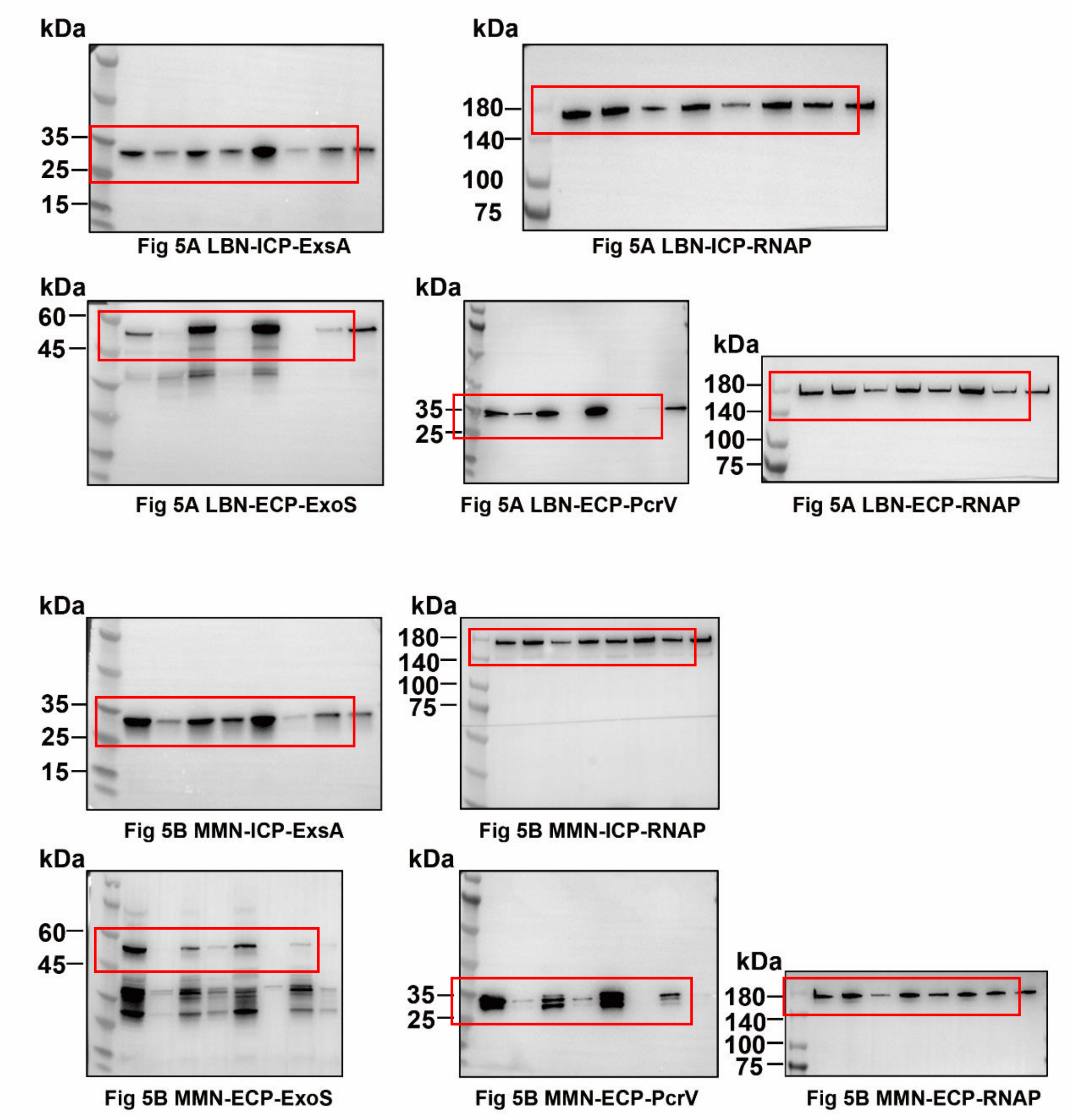

Supplementary Figure S7 Full images of the Western blots used in this study. The magenta frames indicate the cropped parts.
